# Recent Advances in Lotus Seed Starch: Multiscale Structure, Tailored Modification, and Innovative Applications

**DOI:** 10.3390/polym18202543

**Published:** 2026-10-16

**Authors:** Changli Hu, Lu Yuan, Hede Wang, Zhendi Huang, Chuang Liu, Zhexi Weng, Yongyi Tan, Xiaobing Qian, Senwen Deng

**Affiliations:** 1 Guangzhou Restaurant Group Likofu Food Co., Ltd., Guangzhou 511442, China; 2 Hunan Engineering Research Center of Lotus Deep Processing and Nutritional Health Sciences, Hunan Key Laboratory of Economic Crops Genetic Improvement and Integrated Utilization, School of Life and Health Sciences, Hunan University of Science and Technology, Xiangtan 411201, China; yuanlu@mail.hnust.edu.cn; 3 Guangzhou Restaurant Group (Xiangtan) Likofu Food Co., Ltd., Xiangtan 411100, China

**Keywords:** *Nelumbo nucifera*, starch, physicochemical properties, modification technology, industrial application

## Abstract

Lotus seeds represent a valuable botanical resource with dual roles in traditional food and medicinal applications. As the major constituent of lotus seeds, starch possesses unique physicochemical and multiscale structural characteristics that confer significant potential for high-value applications. This review systematically summarizes recent advances in the multiscale structure and physicochemical properties of lotus seed starch. Modification technologies were developed to address the inherent limitations of native lotus seed starch. Existing studies demonstrate that physical, chemical, and enzymatic modifications can precisely tailor the digestive properties, gelation behavior, and processability of lotus seed starch. Furthermore, such modifications have enabled novel applications in fields including prebiotic ingredients, edible packaging, biomedical carriers, and low-GI foods. Future research should prioritize environmentally benign and precisely controlled modification strategies, as well as the elucidation of the material–structure–function relationships, thereby ultimately facilitating the industrialization of novel applications of lotus seed starch. Collectively, these advances are expected to facilitate the high-value utilization of this underutilized starch resource, and to serve as a valuable reference for the broader industrialization of non-conventional starch resources.

## 1. Introduction

Lotus (*Nelumbo nucifera* Gaertn.) is a perennial aquatic herb belonging to the oldest lineages of angiosperms [1]. China is recognized as a major center of origin and a leading producer of lotus [2]. Through long-term natural selection and artificial breeding, lotus has diverged into three primary cultivated types: seed lotus, rhizome lotus, and flower lotus [3,4]. Among them, seed lotus represents an economically significant crop, with large-scale cultivation established in Hunan, Fujian, Zhejiang, and Jiangxi provinces in China, yielding an estimated annual national output of approximately 300,000 tons [1]. Lotus seeds possess a range of pharmacological activities, including antioxidant, anti-inflammatory, neuroprotective, and anti-tumor properties [4,5]. In terms of nutritional composition, dried lotus seeds contain approximately 60–70% total carbohydrates on a dry weight basis, with starch serving as the predominant component, localized mainly in the cotyledons. Lotus seed starch is a key determinant of the texture, flavor, and processing suitability of lotus seed-based products [6].

Lotus seed starch is a typical C-type crystalline starch, characterized by a significantly higher amylose content than conventional cereal starches (e.g., corn and wheat). In addition, its granule morphology and chain length distribution exhibit considerable variability [5,7]. This unique structure profile confers a relatively high gelatinization temperature compared with conventional starches and good gelation capacity. It also imposes inherent limitations, including low swelling power, poor freeze–thaw stability, and a pronounced retrogradation tendency, which is a key factor constraining the shelf life and quality stability of deeply processed lotus seed products [5,7]. Lotus seed starch is non-toxic and biodegradable, and it has demonstrated promising potential for application as a food ingredient at the laboratory scale; however, as a non-conventional starch resource, its full commercial value remains to be exploited.

Previous reviews have concentrated primarily on the fundamental properties and application prospects of lotus seed starch [8,9,10]. In view of the inherent limitations of native starch, various modification strategies, including physical, chemical, biological, and composite modifications, have been employed to tailor its functionality for diverse end uses. Beyond its conventional role in food products, modified lotus seed starch has demonstrated promise in emerging fields, such as packaging films, nanomaterials, and controlled-release systems for bioactive compounds [8,9,10]. These novel research avenues not only expanded the understanding of lotus seed starch’s functional potential, but also established a foundation for its potential high-value utilization, pending validation at pilot and industrial scales.

Against this backdrop, the present review consolidates recent progress in the chemical composition, multiscale structure, and modification technologies of lotus seed starch. By identifying existing research gaps and proposing future directions, this work aims to provide a comprehensive reference for the application of this underutilized, non-conventional starch resources.

## 2. Literature Search Strategy

A systematic literature search was conducted across four electronic databases: Web of Science Core Collection, Scopus, ScienceDirect, and the China National Knowledge Infrastructure (CNKI) for Chinese-language literature, to identify all relevant studies published between January 2005 and June 2026. Supplementary backward citation tracking was performed using Google Scholar and the reference lists of eligible articles. The search terms were combined using Boolean operators as follows: (“lotus seed starch” OR “lotus starch”) AND (“multiscale structure” OR “physical modification” OR “chemical modification” OR “enzymatic modification” OR “dual modification” OR “combined modification” OR “digestibility” OR “resistant starch” OR “application”). Searches were restricted to titles, abstracts, and keywords.

All retrieved records were imported into the reference management software EndNote 21 (Clarivate, Philadelphia, PA, USA); duplicate records were removed both automatically and manually. Subsequently, two independent reviewers screened titles and abstracts to determine initial eligibility. Full texts of potentially eligible articles were subsequently assessed according to the following inclusion criteria: (i) peer-reviewed original research or review articles; (ii) direct relevance to the structure, modification, digestibility, or applications of lotus seed starch; and (iii) provision of experimental data or mechanistic evidence. Primary exclusion criteria were conference abstracts, non-peer-reviewed reports, duplicate publications, and studies not specifically focused on lotus seed starch. Any discrepancies arising during screening were resolved through consensus between the two reviewers.

Exceptions to the above criteria are explicitly identified. First, some Master’s theses were included as grey literature because they provided original experimental data on lotus seed starch that were not available in peer-reviewed sources. Secondly, selected studies on other starches, such as rhizome starch, were not included in the principal literature sample; rather, they were used only for comparative, methodological, or background discussion.

The 96 entries constitute the complete reference list. Of these, 57 form the principal literature sample, i.e., peer-reviewed studies specifically focused on lotus seed starch that were identified by the systematic search; the remaining 39 references were cited for background, comparative, methodological, or regulatory purposes.

## 3. Physical and Chemical Properties of Native Lotus Seed Starch

Lotus seeds are typical high-starch botanical materials, with total carbohydrates accounting for approximately 60–70% of the dry weight, among which starch constitutes the predominant fraction, representing 40–60% of the dry basis. The remaining carbohydrate fraction is composed primarily of non-starch polysaccharides and oligosaccharides. Lotus seeds are characterized by a high amylose content, with the apparent amylose content reaching approximately 45% of total starch [11,12]. This elevated amylose content serves as a critical determinant of intermolecular chain interactions, as the abundant linear chains are capable of forming extensive hydrogen bonds, thereby directing subsequent chain assembly, granular crystallization, and ultimately governing the macroscopic thermal, rheological, and digestive properties of the starch.

The average moisture, lipid, protein, and ash contents of purified lotus seed starch are 8.8%, 0.11%, 0.65%, and 0.06%, respectively, indicating intrinsically low levels of protein and lipid [13]. Notably, the low lipid content reduces the formation of amylose-lipid complexes, thereby preserving the free amylose available for chain reassociation and gelation. In addition, lotus seed starch exhibits relatively low paste transparency and tends to darken during gelatinization, an effect primarily attributable to the oxidation of trace residual phenolic compounds and Maillard reactions involving remnant proteins [14].

**Figure 1:**
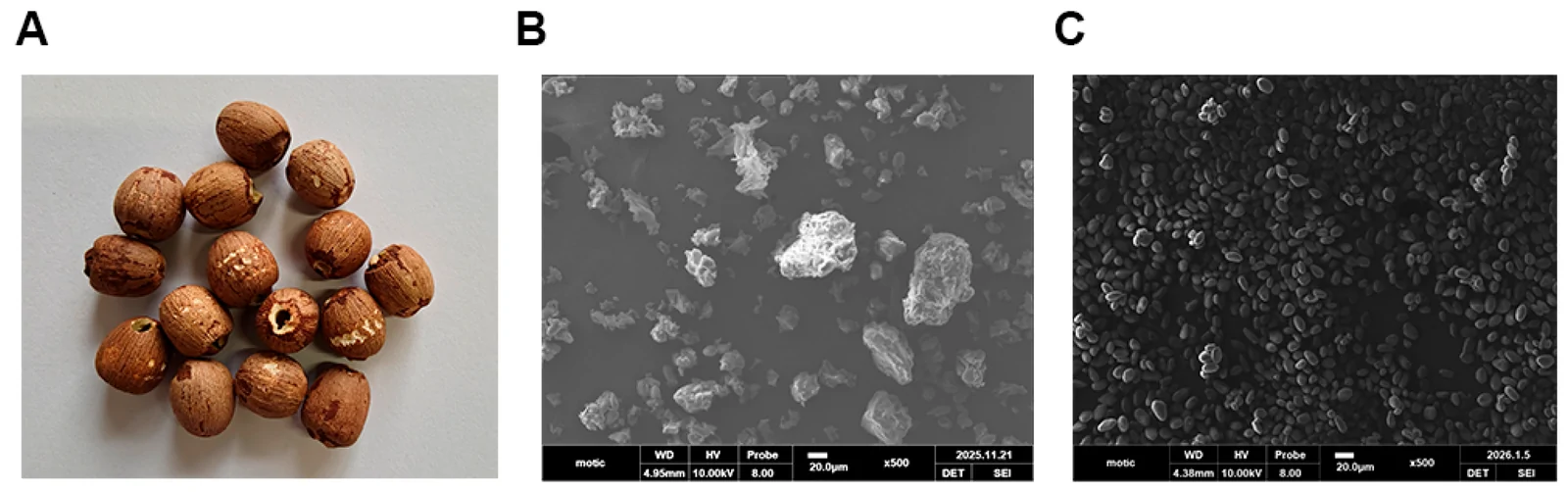
Morphology of lotus seeds, lotus seed flour, and lotus seed starch. (**A**) Lotus seeds; (**B**) SEM image of lotus seed flour; (**C**) SEM image of lotus seed starch.

Reflecting the underlying molecular architecture, lotus seed starch granules are predominantly near-spherical to oval in shape, with smooth surfaces (Figure 1); some granules display a depression or hollow structure at one terminus [11]. Compared with potato and corn starch, lotus seed starch granules are more elongated and exhibit a larger lamellar repeat distance [11]. This smooth, dense granule morphology and enlarged lamellar spacing are direct macroscopic manifestations of tightly packed amylopectin side chains and ordered double-helical arrays within the granular architecture. Under polarized light microscopy, a distinct Maltese cross is observed at the granule center, indicating a well-ordered crystalline structure [15]. These birefringence patterns visually confirm the integrity of radially arranged molecular chains, bridging granular morphology to short-range chain organization and polymorphic crystallinity. Owing to variations in cultivar, extraction procedure, and analytical method, the reported particle size distribution of lotus seed starch varies considerably across studies. Some studies have reported bimodal size distributions [9], whereas a more recent study indicates that most lotus seed starches display a unimodal particle size distribution, predominantly within the range of 2–30 μm [13]. Several factors may account for this discrepancy. First, differences in cultivar and geographical origin may lead to inherent variations in granule size. Second, grain maturity and extraction conditions (e.g., the degree of defatting and deproteinization, and the drying method) can also affect the dispersion state of starch granules and the observed particle size. Furthermore, incomplete protein removal may promote the aggregation of small granules into larger clusters via protein-starch interactions, resulting in an apparently larger particle size and a bimodal distribution. Such protein-mediated aggregation not only alters the granule size distribution but also creates a physical barrier that restricts enzymatic accessibility, directly linking granule morphology to digestive properties [13].

At the level of chain organization, the molecular size distribution of lotus seed starch can be characterized by molecular weight parameters. The weight-average molecular weight (*M*w) and number-average molecular weight (*M*n) of native lotus seed starch, measured by gel permeation chromatography, are 5.36 × 10^6^ Da and 5.08 × 10^6^ Da, respectively [16]. The Z-average radius (*R*z), which describes the spatial extension of starch molecular chains, serves as an indirect indicator of the degree of molecular branching and conformational characteristics. Previous studies have reported polydispersity indices and *R*z values of 3.23 and 168.4 nm, respectively, for native lotus seed starch [17], and 1.28 and 161.5 nm, respectively, in another study [18]. These discrepancies are likely attributable to differences in sample sources and extraction conditions. For example, the former study applied a higher drying temperature and a longer drying period, which could have promoted molecular degradation or aggregation. These molecular parameters, particularly the high *M*w and large *R*z, reflect pronounced chain entanglement and branching.

X-ray diffraction (XRD) analysis serves as a powerful tool for characterizing the crystalline structure of lotus seed starch granules. Native lotus seed starch has been generally classified as a C-type polymorph, predominantly the CA subtype, which exhibits a crystalline pattern approaching that of A-type starch [14]. The diffractogram displays characteristic A-type reflections at 2θ values of 15.1°, 17.0°, 18.0°, and 23.0°, together with a characteristic B-type peak at 2θ = 5.6°, corroborating its classification as a CA-type intermediate polymorph [8]. Lin et al. further reported that the diffraction pattern of lotus seed starch falls between those of typical A-type and B-type starches, thereby reinforcing its categorization as C-type polymorph [16]. In addition, lotus seed starch shows higher relative crystallinity compared with corn and cassava starches [9]. This elevated degree of crystallinity may be attributed to the compact packing of amylopectin double-helices and the extensive hydrogen-bonding network associated with its high amylose content.

Translating crystalline order into macroscopic thermal behavior, starch gelatinization refers to the process in which starch granules absorb water, swell, and lose their crystalline order upon heating in excess water. During gelatinization, water molecules first penetrate the amorphous regions of the granules and initiate swelling. As the temperature rises, hydrogen bonds and van der Waals forces within the crystalline regions are disrupted, resulting in crystal melting. Subsequently, the exposed hydroxyl groups form hydrogen bonds with water, driving further granule expansion. Due to variations in experimental conditions such as temperature, starch concentration, and chemical composition, the reported swelling power and solubility of lotus seed starch vary considerably. For example, at 90 °C, the swelling power of native lotus seed starch ranges from 8.27 to 15.15 g·g^−1^, and its solubility ranges from 6.1% to 31.67% [19,20,21]. Compared with starches from other botanical sources, lotus seed starch exhibits a lower swelling power, which is closely related to its higher amylose content. The abundant amylose chains restrict granule swelling by forming a continuous, rigid network that physically impedes water infiltration and granule expansion. In general, amylopectin content is regarded as the primary factor determining starch swelling, whereas the content and molecular structure of amylose impose constraints on the swelling behavior [21].

Differential scanning calorimetry (DSC) is the primary technique used for evaluating the thermal properties of starch. The gelatinization temperature parameters obtained by DSC reflect the cooking characteristics of starch during heating. For native lotus seed starch measured under high-moisture or excess-water conditions, the onset (*T_o_*), peak (*T_p_*), and conclusion (*T_c_*) gelatinization temperatures generally range from 44.51 to 70.7 °C, 71.56 to 77.73 °C, and 79.16 to 89 °C, respectively, with a gelatinization enthalpy (Δ*H_gel_*) of 4.61–21.2 J/g. Apart from the possible variation in the lotus seed starch substrates, discrepancies in experimental conditions among studies—such as the starch-to-water ratio and the instrument scanning rate—likely constitute a major source of the observed data variability. Notably, the unusually high temperatures of 137.44 °C (*T_o_*), 138.29 °C (*T_p_*), and 143.21 °C (*T_c_*) reported in some studies were obtained from a starch–water suspension containing 70% water [20], and thus should not be viewed as typical gelatinization temperatures under excess-water conditions. Compared with potato and corn starches, lotus seed starch exhibits higher gelatinization temperatures and a wider gelatinization temperature range (Δ*T*), indicating relatively higher thermal stability of its granular structure. These distinctive thermal properties are closely tied to the unique chemical composition and molecular organization of lotus seed starch. The tightly packed structures not only restrict water penetration and granule swelling, but also necessitate greater thermal energy for disruption during gelatinization, manifesting as elevated *T_o_*, *T_p_*, and *T_c_*, along with a higher Δ*H_gel_*. The entangled conformation of amylose chains within the complex granular matrix enhances overall structural cohesion, further stabilizing the granule and accounting for the difficulty encountered in extracting amylose from lotus seed starch. Together, these structural characteristics provide a coherent structural basis for the observed thermal properties and underscore the close structure-property relationships in starch systems.

During a heating-cooling cycle, the gelatinization characteristics of starch largely determine its behavior in cooking and processing. Amylose content, swelling power, lipid content, and amylopectin structure collectively modulate the rheological properties [9]. The pasting curve of lotus seed starch exhibits a pronounced increase in final viscosity upon prolonged heating and subsequent cooling, indicating pronounced retrogradation [13,14]. Ali et al. reported peak, trough, breakdown, final, and setback viscosities of 5314, 3686, 1628, 6758, and 3072 cP, respectively [19]; Wang et al. reported corresponding values of 1842 cP, 1677 cP, 165 cP, 2421 cP, and 743 cP, respectively [21]. These discrepancies likely stem from differences in starch preparation conditions, together with variations in instrumental equipment and experimental parameters. The most pronounced discrepancy lies in breakdown values (1628 cP vs. 165 cP), suggesting considerable differences in starch granule integrity arising from distinct extraction processes. Relative to many other starches, lotus seed starch shows a lower peak viscosity, suggesting restricted granule swelling and a comparatively high gelatinization temperature [13]. The lower peak viscosity, combined with a higher setback viscosity, reflects restricted granule swelling driven by elevated amylose content and extensive chain reassociation during cooling. The elevated setback viscosity further implies a greater tendency for structural hardening or retrogradation upon cooling [21].

At the macroscopic flow level, rheological properties serve to characterize the flow and deformation behavior of materials during processing. The flow curves of lotus seed starch gels are well fitted by the Herschel–Bulkley model [22]. Studies have shown that lotus seed starch gels exhibit concentration- and time-dependent pseudoplastic non-Newtonian behavior [23]. Under the specific experimental conditions, thixotropic behavior is observed at starch concentrations of 2%, 6%, and 8%, whereas antithixotropic behavior occurs at 4%. These shear-thinning and time-dependent behaviors originate from the concentration-dependent entanglement of high-molecular-weight amylose chains, together with the dynamic disruption and re-establishment of hydrogen-bonded associations under shear. The elastic response of lotus seed starch gels is markedly stronger than their viscous response [23]. Their viscoelasticity also exhibits strong concentration dependence: at starch concentrations of 2%, viscoelasticity is negligible, whereas both the storage modulus (G′, elastic modulus) and loss modulus (G″, viscous modulus) increase as the concentration is raised from 4% to 8% [23]. This concentration-dependent viscoelasticity reflects the development of a percolating network of amylose chains and recrystallized double-helical assemblies.

From both digestive and storage standpoints, starch retrogradation refers to the continuous recrystallization of gelatinized starch. During this process, amylose and amylopectin undergo thermally reversible reassociation to form increasingly ordered structures, thereby diminishing the enzymatic digestibility of starch [24]. Retrogradation typically results in quality deterioration of starch-based foods, manifested as shortened shelf life and compromised texture quality; therefore, it is generally considered undesirable in food processing. Due to its relatively high amylose content, lotus seed starch retrogrades more rapidly than some common starches. For instance, the syneresis rate of lotus seed starch gels during retrogradation is higher than that of cassava and corn starch gels [25]. This accelerated retrogradation is intrinsically linked to the elevated amylose content, which furnishes abundant linear chains that facilitate rapid double-helix formation and recrystallization. The retrogradation behavior of lotus seed starch can be regulated by both exogenous and endogenous factors. Hydrocolloids such as xanthan gum, guar gum, and carrageenan have been shown to effectively inhibit its retrogradation [26]. In addition, endogenous polyphenols in lotus seeds (e.g., chlorogenic acid) can markedly alter the retrogradation properties of lotus seed starch gels, reducing retrogradation enthalpy and the overall degree of retrogradation during storage [27].

In summary, native lotus seed starch should be evaluated at least four distinct levels. (i) Granular level: it exhibits relatively higher granular thermal stability, as reflected by elevated gelatinization temperatures (*T_o_*_,_ *T_p_*, *T_c_*_)_ and a relatively high gelatinization enthalpy (Δ*H_gel_*), which arise from its dense crystalline architecture and high amylose content [9,13]. (ii) Paste level: however, it shows limited paste stability under heating and shear, and its paste exhibits non-Newtonian, shear-thinning (pseudoplastic), and thixotropic behavior in a concentration-dependent manner [23]. (iii) Gel level: native lotus seed starch can form gels upon cooling, but the gel strength is relatively weak and varies with starch composition and molecular structure [19,23]. (iv) Storage level: the resulting paste/gel has poor storage stability, as evidenced by a pronounced retrogradation tendency and poor freeze–thaw stability [28,29].

## 4. Advances in Lotus Seed Starch Modification

Although native lotus seed starch possesses distinctive physiological functionalities attributable to its unique C-type crystalline structure and high amylose content, these structural attributes also confer notable processing constraints. In terms of thermal properties, it exhibits a relatively high gelatinization temperature. Regarding rheological properties, it displays weak gel strength and inadequate shear resistance. With respect to processing stability, lotus seed starch is susceptible to retrogradation and demonstrates poor freeze–thaw stability. Functionally, it has limited cold-water solubility and negligible emulsifying activity [9].

Among these limitations, starch aging and retrogradation constitute the most critical impediments to the practical application of lotus seed starch. The recrystallization of starch molecules disrupts three-dimensional network architecture established via starch–protein interactions, resulting in diminished water-holding capacity, textural hardening, undesirable coarseness, and shortened shelf life. Collectively, these deteriorative changes severely constrain the large-scale utilization of lotus seed starch in premium food applications [9].

**Figure 2:**
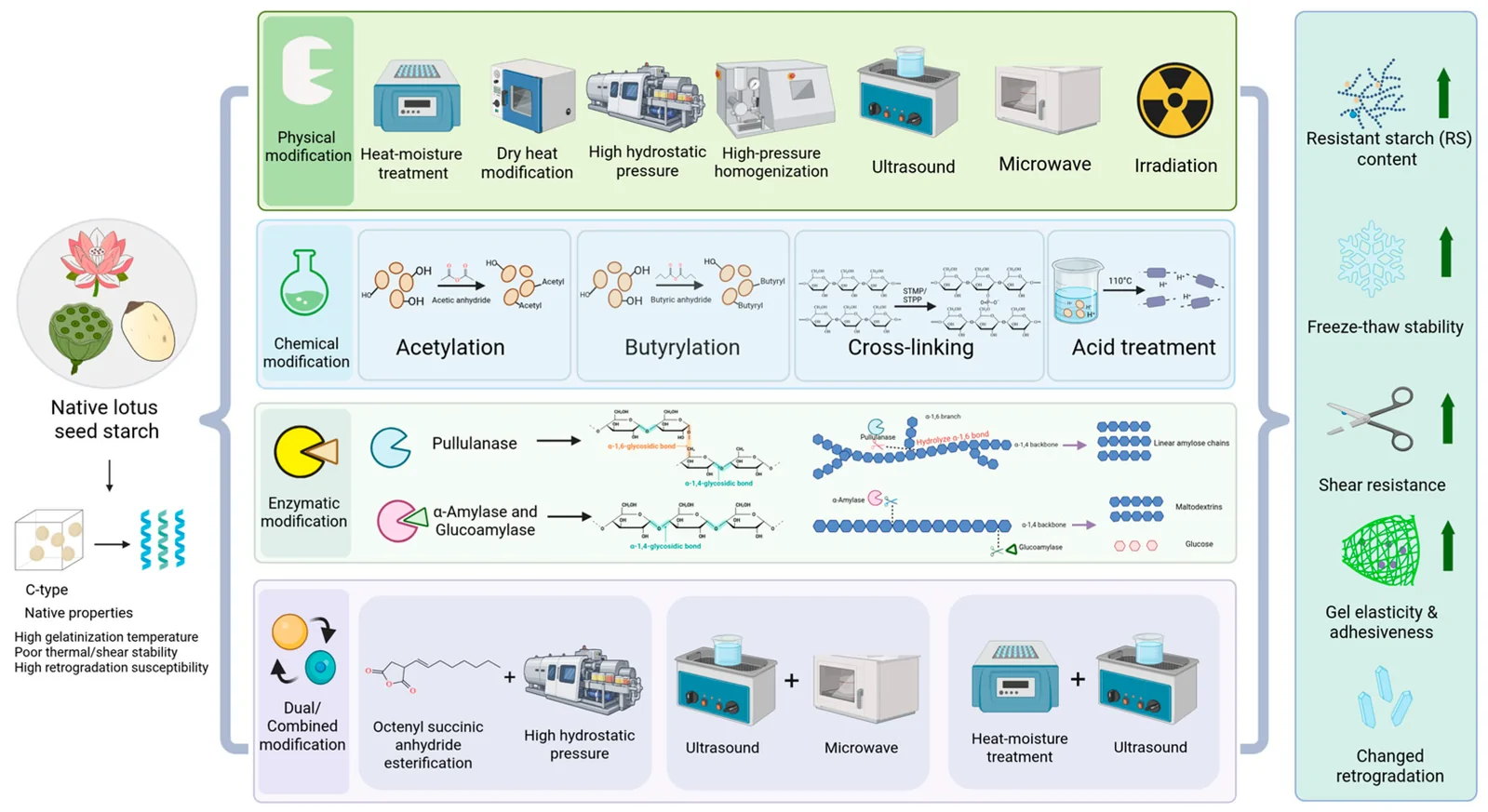
Major modification methods for lotus seed starch.

To mitigate these processing constraints, starch modification technologies have been extensively employed to reorganize the multiscale architecture of lotus seed starch (Figure 2). Current modification strategies can be broadly categorized into four types: physical, chemical, biological, and composite modification (Table 1). Among them, composite modification, which integrates the complementary advantages of distinct techniques (e.g., physical field-assisted chemical cross-linking), enables precise regulation of starch chain conformation and aggregate behavior, and has thus emerged as a prominent research focus in recent years [30].

Regarding the modification substrate, isolated starch is predominantly used as the raw material, whereas a minority of studies employ whole flour (Table 1). Whole flour confers cost advantages but exhibits greater compositional complexity and inferior uniformity. Its non-starch components may interfere with the modification process through complexation, energy absorption, or free-radical scavenging, thereby rendering the modification outcomes less predictable than those achieved with isolated starch. Conversely, isolated starch ensures better batch-to-batch reproducibility and is better suited for high-value applications.

Beyond conventional applications, modified lotus seed starches can function as stabilizers, thickeners, fillers, and gel matrices, with broad applications in low-fat meat products, bakery products, and functional food formulations, thereby substantially increasing the added value of lotus seed resources.

### 4.1. Physical Modification

Physical modification technologies respond to the food industry’s growing demand for “clean label” formulations and environmentally conscious manufacturing, given that they typically obviate the need for chemical reagents and exhibit a higher degree of biosafety (Table 1) [30]. However, it should be critically acknowledged that physical treatments are not inherently green or environmentally preferable. Their core advantage resides in the multi-scale reorganization of starch—encompassing molecular chain conformation, granular microstructure, and mesoscopic aggregate architecture—through modulation of physical field parameters such as temperature, pressure, shear force, and electromagnetic irradiation, thereby enabling targeted optimization of gelatinization behavior, thermal stability (relative to native starch), and retrogradation resistance [30]. Nevertheless, the environmental and economic sustainability of these methods warrants holistic assessment, considering not only energy demand, equipment costs, and scalability constraints, but also solvent use in certain procedures.

The industrial readiness of physical modification methods varies considerably. In particular, HMT is commercially established, while high hydrostatic pressure (HHP, also referred to as high-pressure processing (HPP) in some studies), ultrasound, and microwave-assisted approaches for starch modification remain at an early developmental stage, constrained by batch-mode operation, nonlinear acoustic scale-up, high energy intensity, and limited penetration depth. Consequently, these energy-intensive technologies are economically viable only for high-value output products and remain largely confined to pilot-scale demonstrations and niche commercial applications.

Based on whether substantial thermal effects are involved, physical modification can be classified into conventional thermal processing and emerging non-thermal processing. Conventional thermal treatments (e.g., hydrothermal treatment and extrusion) are industrially mature, but high temperatures induce thermal degradation of heat-sensitive bioactive compounds (such as polyphenols and flavonoids) in lotus seed starch and may trigger Maillard reactions, consequently compromising product color and flavor. In contrast, non-thermal processing technologies operate under ambient or mild low-temperature conditions to realize starch structural modification via physical fields while preserving the inherent color and organoleptic properties of the final product [30].

Currently, non-thermal technologies such as HHP, ultrasound, and irradiation have been extensively applied in starch modification research [41]. Their underlying mechanisms primarily involve cavitation, mechanical shear, and isostatic compression, which induce disentanglement, rearrangement, or partial melting of starch crystallites. These structural changes could enhance starch solubility and gel properties, while change the enzymatic digestibility behavior of starch. Owing to its exceptional capacity to preserve the natural nutritional and sensory quality of foods, non-thermal physical modification has emerged as one of the most promising strategies for the high-value utilization of lotus seed starch, pending scale-up validation.

#### 4.1.1. Thermal Physical Modification

Thermal physical modification, which uses thermal energy to promote the mobility of starch molecular chains, represents an efficient approach for in situ starch modification. Heat-moisture treatment (HMT), a classic thermophysical process, relies on thermal activation under restricted moisture to promote the melting and rearrangement of crystalline regions without disrupting granule integrity [31,42]. A major industrial advantage of HMT is that it obviates the need for prior starch extraction and can be applied directly to whole lotus seed flour, thereby streamlining the processing workflow and reducing production cost [31]. During HMT, the formation of amylose-lipid complexes and the ordered lateral stacking of double helices markedly restrict molecular mobility in amorphous regions, thereby considerably enhancing the thermal stability of lotus seed starch. After modification, the *T_o_*, *T_p_*, *T_c_*, and Δ*H_gel_* of lotus seed starch increase to 57.50 °C, 87.26 °C, 108.25 °C, and 16.22 J/g, respectively, all significantly higher than the corresponding values of native starch [19]. Structural characterization further reveals that although HMT retains the C-type crystallinity of lotus seed starch, the relative crystallinity and double-helix content are markedly increased, indicative of denser granular packing. Macroscopically, these changes manifest as reduced swelling capacity, enhanced solubility, and diminished gel hardness [19].

Unlike HMT, dry heat modification (DHM) is conducted in the absence of added moisture and relies on solid-phase reactions to drive molecular reorganization. Single DHM promotes the formation of double-helical microcrystals, leading to higher pasting temperature and reduced setback value [32]. This phenomenon could be attributed to the enhanced intermolecular interactions induced by DHM, which necessitates greater external energy to disrupt. However, when hydrophilic colloids (such as sodium alginate) are introduced for synergistic DHM, the system undergoes distinct physicochemical transformations: exogenous macromolecules interact with starch chains via esterification or cross-linking, thereby perturbing the integrity of the original crystalline network. This combined strategy improves cold stability and anti-sedimentation properties of starch. More importantly, it significantly enhances the mechanical strength and moisture-barrier performance of starch-based films, offering new opportunities for applying lotus seed starch in food packaging materials [32].

#### 4.1.2. Microwave Modification

Microwave technology operates via electromagnetic radiation in the frequency range of 3–30 GHz, enabling rapid internal volumetric heating of materials through dipole rotation and ionic conduction, thereby constituting a representative thermophysical processing technique in the food industry. For high-amylose, highly crystalline lotus seed starch, microwave modification elicits not only thermal effects but also non-thermal effects that induce vibrational perturbations and conformational rearrangement of starch molecular chains, thereby substantially reshaping its multiscale structure and digestibility profile [43].

Zeng et al. (2016) [17] demonstrated the modulatory effect of microwave power density on starch conformation. Across the power range of 2.4–8.0 W/g, increasing microwave energy input resulted in progressive reductions in swelling power, amylose leaching, and radius of gyration, as well as a decrease in the branching degree and a lower proportion of long branch chains of lotus seed starch. Microwave treatment can also facilitate amylose-amylose interactions. The reduction in amylose leaching, coupled with enhanced starch–water interactions, not only modulated the physicochemical properties of the gel but also affected the digestive properties of microwave-gelatinized lotus seed starch in vitro. This “molecular chain contraction” effect markedly reduces α-amylase accessibility, resulting in concurrent increases in RS and slowly digestible starch (SDS) contents and consequently lower hydrolysis index (HI) and estimated glycemic index (eGI) values. It should be noted that the reduced GI values were estimated from in vitro starch hydrolysis, not measured in humans. These findings provide a theoretical basis for the development of low-GI lotus seed-derived products, but human studies are needed to confirm the effects.

Microwave treatment also alters particle morphology and crystalline configuration. Nawaz et al. (2018) [44] reported that, with treatment durations extending from 1 to 5 min, prolonged microwave exposure induces a “micro-nanization” effect on starch granules: the average particle size decreases, whereas specific surface area and pore volume increase significantly. At the crystalline level, high-energy microwave radiation induces partial melting and recrystallization of the characteristic C-type crystallinity of lotus seed starch, promoting its conversion into CA- or CB-type mixed crystal forms. This multiscale structural evolution enhances the interfacial activity of the modified starch, improving its oil absorption, emulsion stability, and water-holding capacity, thereby broadening its application potential as a functional food ingredient.

It is noteworthy that the aforementioned microwave treatments targeted extracted lotus seed starch and lotus seed flour, respectively. Meanwhile, the microwave treatment conditions also varied between these studies. Future studies should standardize the reporting of cultivar, extraction method, amylose content, and precise microwave processing parameters to facilitate meaningful cross-study comparisons.

#### 4.1.3. High-Pressure Modification

High-pressure modification technology exploits the unique effects of pressure fields, together with accompanying shear and cavitation forces in dynamic modes, to overcome the thermodynamic limitations of conventional thermal processing, thereby establishing it as an important technology for the valorization of starch products [45]. This technological system is generally divided into two categories: HHP and dynamic high pressure (DHP), with the latter typically exemplified by high-pressure homogenization (HPH). Although both methods utilize pressure energy, they differ substantially in their underlying mechanisms and resultant structural effects.

HHP applies a static, uniformly distributed pressure to the material under ambient or low-temperature conditions. Its principal advantage lies in the instantaneous and homogeneous transmission of pressure devoid of temperature gradients, which weakens intramolecular hydrogen-bond networks and induces an “orderly dissociation-rearrangement-recombination” of starch chains while preserving granular integrity [45]. Studies on lotus seed starch have shown that its native C-type crystallinity undergoes a pressure-induced phase transition, progressively converting into a more compact B-type form. As pressure increases (up to 600 MPa), relative crystallinity declines, and granules swell due to water uptake, leading to increased particle size and a broader size distribution [46]. Rheological measurements further reveal that under moderate pressure (≤500 MPa), the mobility of starch chain segments is restricted, resulting in increased G′ and G″ and the formation of a dense network. Conversely, excessive pressure (600 MPa) induces complete gelatinization and depolymerization of molecular chains, leading to a reduction in moduli. Moreover, HHP decreases Δ*H_gel_* and retrogradation values, signifying potent inhibition of short-term retrogradation and improved thermal and storage stability [28].

In contrast, HPH propels starch suspensions through narrow valves at high pressure (up to 180 MPa), generating intense shear, impact, and cavitation effects. The high mechanical energy causes physical fragmentation of lotus seed starch granules, which display an irregular “broken porcelain-like” morphology and a broadened particle size distribution [33]. Structural analyses show that HPH facilitates the transition from C-type to B-type crystallinity, while reducing short-range molecular order. Due to the loss of granular integrity, the swelling power and solubility of the modified starch increase, gelatinization enthalpy decreases, and the in vitro digestibility is enhanced, primarily attributable to the enlarged accessible surface area for enzymatic attack [33].

Taken together, high-pressure modification obviates the need for chemical reagents, avoids the formation of thermal by-products (e.g., Maillard reaction products), and preserves the intrinsic quality attributes of foods [45]. By precise modulation of pressure parameters, lotus seed starch can be deliberately engineered to shift from an enzyme-resistant profile toward a rapidly digestible one, and from pronounced retrogradation tendency toward enhanced storage stability.

#### 4.1.4. Other Physical Modification Methods

Ionizing radiation (including γ-rays and electron beams) is a non-thermal processing technology that not only effectively inactivates pests and microorganisms, but also cleaves starch molecular chains via high-energy particle bombardment, thereby modifying its multiscale architecture [47]. Studies have shown that, after irradiation at doses ≤ 30 kGy, the apparent total starch content of lotus seed increases, which can be attributed to the formation of low-molecular-weight fragments resulting from molecular degradation [48]. When lotus seed starch is irradiated with γ-rays at different doses (5, 10, 15, and 20 kGy), granular morphological alterations in starch granules become more pronounced with increasing dose, mainly manifested as increased surface roughness, while the C-type crystalline pattern remains unchanged. Irradiation impairs the pasting properties, reduces gelatinization temperature and relative crystallinity, and decreases amylose content [20]. These results indicate that irradiation is more suitable for producing low-viscosity, rapidly digestible lotus seed starch-based ingredients.

Ultrasonic treatment (US) exploits the cavitation effect generated by high-frequency sound waves (20 kHz–10 MHz) to physically erode starch granules through microjets and shock waves. For native lotus seed starch, US (20 kHz, 170 W) exerts a distinct activating effect. Ultrasonication increases the swelling power (17.67 g/g) and solubility (17.90%) of lotus seed starch, while enhancing its thermal stability compared with native starch under the tested conditions, as evidenced by increased Δ*H_gel_* [34]. The underlying mechanism is presumably that ultrasonic treatment disrupts granule surfaces and amorphous regions via the cavitation effect, leading to increased swelling and solubility; concurrently, it promotes the reassembly of fragmented molecular chains, forming a certain type of denser double-helical structure [34]. For debranched V-type lotus seed starch (VLS), US at an appropriate power (e.g., 360 W) does not disrupt V-type crystallites; instead, it improves short-range molecular order and crystallinity. Ultrasonication results in a denser gel network, which substantially increases resistance to digestive enzymes (higher RS content) and enables the encapsulation of functional molecules, such as butyric acid, within the hydrophobic cavities of V-type structures [49].

### 4.2. Chemical Modification

Chemical modification is a core strategy for overcoming the processing bottlenecks of native lotus seed starch, particularly in applications that require fundamental changes in starch charge properties or substantial enhancement of water-holding capacity.

Owing to the inherent limitations of native lotus seed starch, including limited paste stability under heating and shear, a high tendency toward retrogradation, and weak gel strength, chemical modification enables the design of starch functionality through the targeted regulation of the chemical configuration and supramolecular aggregation structure of molecular chains [30]. Based on reaction mechanisms and changes in molecular structure, chemical modification of lotus seed starch can be broadly classified into two categories: degradative modification and functional derivatization.

Degradative modification can improve the rheological properties and solubility of starch primarily by cleaving glycosidic bonds and reducing the degree of polymerization (DP), with acid hydrolysis and oxidative degradation being typical approaches. Functional derivatization reshapes starch physicochemical properties by introducing new chemical groups onto the starch backbone or by forming intermolecular covalent bonds. Through reactions such as cross-linking, esterification, and etherification, new functional groups (e.g., carboxyl and acetyl groups) are introduced, or covalent cross-linked networks are constructed, thereby imparting unique functional attributes.

Chemical modification can significantly alter the physicochemical properties of lotus seed starch at multiple scales—molecular, crystalline, and granular (Table 1). The modification outcome is strongly dependent on the degree of substitution (DS), reaction medium, and process parameters (e.g., pH, temperature, reaction time), and is also closely related to the botanical source, molecular structure, and granular characteristics of lotus seed starch [9].

#### 4.2.1. Esterification Modification

Esterification modification involves the covalent introduction of acyl groups (e.g., acetyl, butyryl, octenylsuccinyl) into the hydroxyl sites of starch molecules through the formation of ester bonds. By incorporating hydrophobic acyl chains onto starch chains, acylation reshapes the intermolecular hydrogen-bond network, supramolecular aggregation structure, and surface hydrophobicity of native starch at multiple scales. Consequently, modified starch acquires a range of distinct functional properties: core processing attributes such as gelatinization behavior, rheological properties, and resistance to retrogradation are markedly improved, and its digestive characteristics can be precisely tailored [30]. To date, studies on the esterification of lotus seed starch have mainly focused on three types of acylation: octenyl succinic anhydride (OSA) modification, acetylation, and butyrylation.

OSA-modified starch (OSA starch) is an amphiphilic polymer obtained via the esterification of starch with OSA in a weakly alkaline aqueous medium. Studies on OSA-modified lotus seed starch have shown that with increasing OSA dosage, the DS rises from 0.0148 to 0.0389, while the relative crystallinity gradually decreases due to amorphous regions of the starch being replaced by OSA reagent [50]. Concurrently, the average granule size increases due to aggregation induced by the hydrophobic surface layer formed during modification. The esterification reaction occurs preferentially within the amorphous regions of the starch granules, and the introduced octenyl chains disrupt the intermolecular hydrogen-bond network via steric hindrance. This structural alteration endows the modified starch with better interfacial activity. When employed to encapsulate functional lipids such as conjugated linoleic acid (CLA), OSA-modified lotus seed starch achieves an inclusion complex index of up to 82.3%, substantially higher than that of native starch, which is attributed to strengthened hydrophobic interactions between the starch and lipids [50].

Acetylation is a typical chemical modification strategy for introducing acetyl groups and weakening intermolecular hydrogen-bond within starch. Lin (2024) [35] prepared a series of acetylated lotus seed starches using an aqueous reaction system. The DS of the acetylated products increased linearly with the amount of acetic anhydride added. The introduction of acetyl groups did not alter the C-type crystalline pattern of lotus seed starch but significantly reduced its relative crystallinity and gelatinization enthalpy. The steric hindrance effect of acetyl groups effectively suppresses amylose/amylopectin chain reassociation, resulting in markedly improved transparency, swelling power, and solubility compared with native starch, accompanied by a reduced gelatinization temperature. These alterations effectively mitigate the inherent drawbacks of native lotus seed starch, including its limited transparency and elevated gelatinization temperature.

Unlike acetylation, the introduction of butyryl groups induces more profound structural reorganization in lotus seed starch. Li et al. (2022) [36] prepared butyrylated lotus seed starches (LSB) with different DS values via an aqueous reaction system. Beyond a critical butyric anhydride dosage, the reaction plateaued, exhibiting no significant regioselectivity at the C2, C3, or C6 hydroxyl positions. Butyrylation induced surface erosion and pitting on the granules and triggered a polymorphic transition from C-type to V-type crystallinity. This transition was accompanied by more compact molecular chain packing and a substantial elevation in RS content, with RS accounting for up to 53% of total starch. These findings indicate that butyrylation serves as an effective strategy for developing high-RS functional ingredients.

#### 4.2.2. Crosslinking Modification

Cross-linking represents a pivotal approach for improving the stress resistance and processing stability of starch. This technique utilizes polyfunctional cross-linking agents (e.g., sodium trimetaphosphate (STMP), sodium tripolyphosphate (STPP), epichlorohydrin) to establish stable covalent interchain bridges, thereby modifying the supramolecular assembly and surface characteristics of starch granules [51]. In high-amylose lotus seed starch, cross-linking preferentially occurs in amorphous regions. The resulting covalent network substantially restricts the segmental mobility of starch chains, inducing fundamental alterations in thermal and rheological behaviors. Functioning as “molecular rivets,” cross-linking bonds inhibit excessive swelling of starch granules, manifested by significantly elevated *T_o_*, *T_p_*, and *T_c_* (gelatinization temperatures) and a concomitant reduction in Δ*H_gel_*, which is attributed to restricted water penetration and incomplete crystalline melting [37]. The compact cross-linked network yields considerably diminished solubility and swelling power relative to native starch [52]. Furthermore, this physical barrier effectively constrains digestive enzyme access to starch chains, resulting in a positive correlation between RS content and the degree of cross-linking [37].

#### 4.2.3. Acid Treatment

Acid hydrolysis represents one of the most established techniques for the chemical modification of starch and is generally performed at room temperature or under gelatinization-inhibiting conditions. Its mechanism entails proton (H^+^) attack on glycosidic bonds, inducing bond scission and enabling precise control of molecular weight and crystallinity [53]. Compared with root and tuber starches, lotus seed starch exhibits notable resistance to acid hydrolysis, mainly attributable to its relatively high amylose content and distinctive C-type crystalline structure. This compact granular network effectively impedes the infiltration of proton (H^+^) into the granule interior and the ensuing cleavage of glycosidic bonds. As a result, acid treatment of lotus seed starch typically demands tighter control of reaction parameters (such as acid type, concentration, temperature, and duration) [54].

Among the various acid reagents, citric acid (CA)—an organic acid that serves as a natural intermediate in the tricarboxylic acid (TCA) cycle and is classified as Generally Recognized as Safe (GRAS) substance—is a promising modifier. Unlike inorganic acids, CA not only provides protons but also introduces steric hindrance through its three-carboxyl-group structure, thereby moderating the hydrolysis process. Ali et al. (2020) [19] demonstrated that, compared with native lotus seed starch, CA treatment enhances the thermal stability of lotus seed starch, as evidenced by concentration-dependent elevations in *T_o_*, *T_p_*, and *T_c_* (gelatinization temperatures), as well as Δ*H_gel_*. XRD and Fourier-transform infrared (FTIR) spectroscopy analysis indicate that acid hydrolysis promotes the disentanglement and reorganization of molecular chains, leading to an increased degree of molecular order and a higher proportion of double-helix structures. In addition, moderate acid hydrolysis selectively removes the amorphous surface layer and modifies the surface hydrophilicity of granules, resulting in increased solubility and enhanced gel elasticity and adhesiveness. Overall, CA treatment substantially improves the physicochemical and thermal properties of lotus seed starch, supporting its potential use as a gelling agent, thickener, and stabilizer.

Despite the widespread commercialization of chemically modified starches, these products are subject to stringent regulation as food additives. In the United States, starch modified with STMP or STMP/STPP is permitted, with residual phosphate restricted to 0.04% P for STMP alone and 0.40% P for STMP/STPP combinations [55]. The European Food Safety Authority (EFSA) reevaluated phosphate-modified starches and assigned an ADI of “not specified”, relying on read-across toxicology, in silico non-genotoxicity, rat studies up to 31,000 mg/kg body weight/day with no treatment-related effects, and human tolerance data [56]. Likewise, the joint FAO/WHO Expert Committee on Food Additives (JECFA) and the Scientific Committee on Food (SCF) granted an ADI of “not specified” for modified starches and identified no reproductive or long-term concerns for phosphated distarch phosphate [57]. Esterified derivatives (e.g., OSA starch) are additionally supported by JECFA evaluations [58].

### 4.3. Enzymatic Modification

Enzymatic modification, characterized by mild reaction conditions, remarkable catalytic specificity, and the production of innocuous, safe products, is progressively recognized as a viable alternative to traditional physical and chemical approaches (Table 1). This technique conforms to the industrial demand for green food manufacturing and sustainable development [59]. Attributed to the high amylose content and high crystallinity of lotus seed starch, enzymatic modification typically achieves structural remodeling through two main strategies: debranching and pore formation.

Pullulanase specifically hydrolyzes α-1,6 glycosidic bonds in amylopectin, removing branched structures and generating linear amylose chains. Studies utilizing lotus seed flour have shown that pullulanase treatment significantly increases the amylose and RS contents. The debranching activity of pullulanase disrupts the tree-like architecture of amylopectin and reduces intermolecular entanglement and resistance to recrystallization, thereby accelerating gelatinization and markedly improving the viscosity and thickening properties of the gelatinized system [31].

Synergistic action of α-amylase and glucoamylase enables the fabrication of a novel porous lotus seed starch characterized by a well-developed pore structure while preserving its C-type crystallinity. The optimal concentration of the enzyme mixture was determined to be 1.5% (*w*/*w*, based on starch dry weight). Under this condition, the resulting pores on the granule displayed uniform dimensions and good interconnectivity, and the water absorption capacity, oil absorption capacity, and specific surface area attained their maximum values. The enzymatic hydrolysis exhibited spatial specificity, preferentially attacking the amorphous regions. Consequently, the average granule size diminished due to surface etching, while the relative crystallinity, molecular order, and bound water content rose, accompanied by more compact packing of semicrystalline lamellae. This unique combination of a porous morphology and higher crystallinity substantially retarded the rate of enzymatic hydrolysis, leading to concurrent increases in SDS and RS contents [38].

### 4.4. Dual Modification

Dual modification integrates two distinct techniques (e.g., physical-chemical or physical-physical combinations) and exploits their synergistic effects to overcome the inherent limitations of single-modification approaches (Table 1). In the case of lotus seed starch, this strategy not only achieves retrogradation resistance, thermal stability, and shear resistance that are difficult to achieve via single-modification method, but also reduces energy consumption through process complementarity [30].

The coupling of HHP with esterification serves as a representative physical-chemical dual modification strategy. HHP modulates the structural compactness of starch granules, thereby governing the efficacy of subsequent chemical modification. Sun et al. (2020) [50] adopted a dual modification approach that integrated HHP pretreatment and OSA esterification. Compared with single OSA esterification, HHP pretreatment induced densification of the granular structure and significantly diminished the number of accessible hydroxyl sites available for OSA substitution. Specifically, at an OSA dosage of 9%, the DS of the dual-modified sample (H-OSA-9%) decreased substantially from 0.0389 to 0.0125, corresponding to a 67.9% reduction. Despite this low DS, the HHP-induced increase in amylose leaching endowed H-OSA samples with better interfacial activity and emulsion stability, underscoring the regulatory role in tailoring the microstructure of chemically modified starch [39].

In addition to physical-chemical dual modification, physical-physical synergistic modification has also garnered increasing research interest. Zhao et al. (2019) [40] treated lotus seed starch–green tea polyphenol (LS-GTP) complexes with combined ultrasound (200–1000 W) and microwave (150–225 W) treatment. Ultrasound-microwave (UM) technology, through the synergistic “cavitation-thermal” effect, not only mitigates the energy input required for microwave-only processing, but also significantly increases the complexation yield. The underlying mechanism is that microjets generated by ultrasonic cavitation accelerate the diffusion of polyphenols into the amorphous regions of starch. Notably, the applied power level critically governs the structural mode of complexation: at low power, non-inclusion complexes are predominantly stabilized via hydrogen bonding, whereas elevated power levels favor the formation of V-type inclusion complexes [40].

Chandak et al. (2024) [34] reported that, compared with single-modification treatments, the combined application of HMT and US induces more pronounced granular etching, characterized by surface roughening, cracking, and eventual fragmentation. Such extensive structural disruption profoundly modulates the digestibility profile: dual modification elevates RS content while concomitantly reducing SDS content. A plausible explanation is that the fractions originally assigned to the SDS pool primarily reside in moderately ordered amorphous or paracrystalline regions. Upon combined HMT-US treatment, these regions undergo chain scission followed by retrogradation, culminating in the formation of ordered structures that exhibit pronounced resistance to enzymatic hydrolysis. Furthermore, the order in which modifications are applied exerts a decisive influence on rheological behavior, underscoring the need for future studies to elucidate how processing sequence directs the assembly of gel network architectures.

## 5. Innovative Applications of Lotus Seed Starch

**Figure 3:**
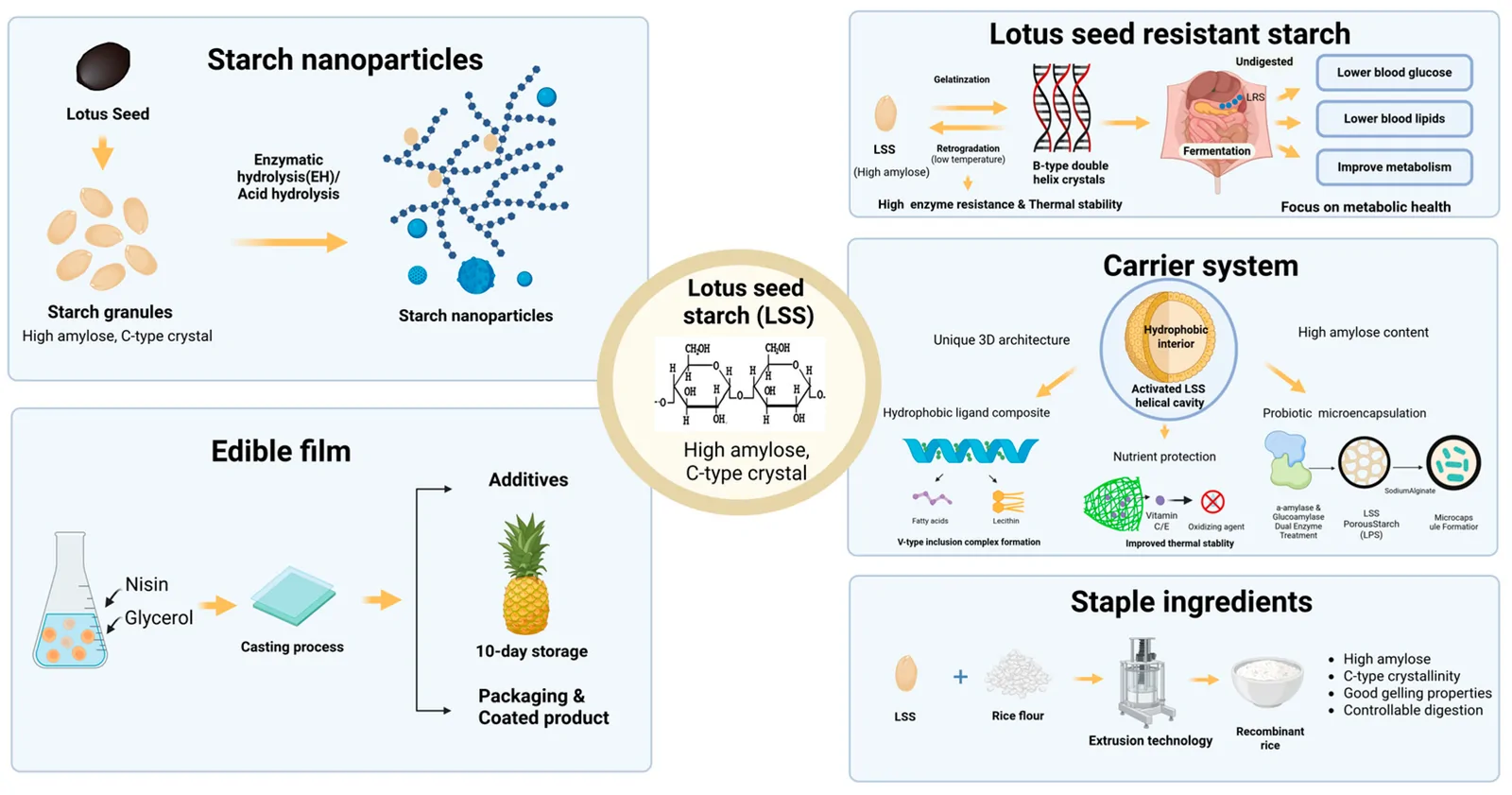
Schematic summary of the innovative applications of lotus seed starch.

Lotus seed starch, characterized by high amylose content, elevated gelatinization temperature, and accelerated retrogradation, constitutes a promising functional starch resource. Its functional properties can be further tailored via physical (e.g., microwave, HHP, HMT), chemical (e.g., esterification, cross-linking), and enzymatic modification. At present, it has been employed in packaging films, nanomaterials, low-GI functional foods, and controlled-release delivery systems for bioactive compounds, demonstrating substantial potential for industrial application (Figure 3).

### 5.1. Edible Coatings/Films

Polysaccharide-based packaging films have emerged as one of the most promising alternatives to petroleum-derived plastics, owing to their biodegradability, renewability, and intrinsic film-forming capacity [60]. Against this backdrop, lotus seed starch constitutes a promising alternative for biodegradable coatings and films, mainly due to its high amylose content, which favors film formation [9].

Solution casting is the mainstream method for fabricating starch-based films. Lin et al. (2011) [61] developed a lotus seed starch coating containing 0.02% (*w*/*w*) nisin and 1.5% (*w*/*w*) glycerol and applied it to the preservation of fresh-cut pineapple. When combined with polyethylene packaging, a 2% or 3% (*w*/*w*) starch coating maintained the total microbial count below 1 × 10^5^ CFU/g over 10 days of ambient storage, and inhibited fruit browning and softening [61]. These pioneering studies established the feasibility of lotus seed starch as a film-forming polysaccharide; however, as noted in the recent literature, native starch films generally suffer from comparatively unfavorable mechanical properties and relatively high water vapor permeability arising from their inherently loose, hydrophilic network [60].

Physical and chemical modifications can improve the mechanical properties and barrier performance of starch films. Zhang et al. (2017) [62] further optimized the film-forming process of dry-heat-modified lotus seed starch, identifying a starch concentration of 3.53% (*w*/*w*), a glycerol content of 1.51% (*w*/*w*), and a drying temperature of 80.61 °C as optimal conditions. Blending heat-moisture-treated lotus seed starch (HMLS) with carboxymethyl cellulose (CMC), especially with the addition of red turnip peel extract (RTE), yields an HMLS-RTE composite film with improved thermal stability, mechanical properties, and moisture barrier properties relative to native starch films, functioning as an intelligent packaging material with pH-responsive behavior [63]. The enhanced performance is mechanistically attributed to HMT-induced increases in relative crystallinity and promotion of amylose chain reassociation, while CMC introduces additional carboxyl groups capable of forming hydrogen bonds and electrostatic interactions with starch chains. Collectively, these effects reinforce the film network and reduce the free volume available for water diffusion [63]. Cross-linking is another effective reinforcement strategy. Using STMP, Chandak et al. (2022) [52] prepared cross-linked lotus seed starch films (CL-LSFs), which exhibited reduced water vapor permeability without altering film thickness and achieved a tensile strength of 12.52 MPa. The introduction of covalent intermolecular bridges restricts polymer chain mobility and increases network density, thereby effectively overcoming the low mechanical strength and limited barrier performance of native starch films.

The incorporation of nanofillers or other biomacromolecules into lotus seed starch matrices can impart active functionalities, including antioxidant and antimicrobial activities. Deng et al. (2024) [64] developed a lotus seed core powder starch (LCPS)-based film containing cellulose nanocrystals (CNCs) and grapefruit essential oil Pickering emulsions. Benefiting from the regular morphology and high amylose content of LCPS granules, the composite film exhibited a denser structure, enhanced inhibitory effects against *Escherichia coli* and *Staphylococcus aureus*, and extended the shelf life of chilled pork. Similarly, a coating formulated from sesame protein isolate and lotus seed starch, combined with *Garcinia indica* extract, was developed by Sharma et al. (2022). This composite coating maintained sapodilla firmness through synergistic effects and extended its shelf life to 12 days, demonstrating practical value in tropical fruit preservation [65].

Although these studies confirm the feasibility of lotus seed starch-based materials, this research area remains comparatively nascent relative to more established polysaccharide film systems [60]. Further investigations are needed in areas such as precise structural design, multiscale interfacial compatibility, and continuous film-forming processes. For example, a biodegradable composite film prepared from lotus root starch, whey protein concentrate, and psyllium husk exhibits enhanced physicochemical, barrier, optical, mechanical, thermal, and surface properties compared with films based solely on lotus root starch [66]. Future efforts should focus on developing lotus seed starch-based films with intelligent functionalities (e.g., freshness indicators) and high barrier performance, which will be pivotal for translating laboratory research into industrial-scale applications.

### 5.2. Lotus Seed Starch Nanomaterials

In recent decades, the development of bio-based materials with tailored properties via nanotechnology has become an important research direction in materials science and green chemistry. As a natural polysaccharide with wide availability, biocompatibility, and biodegradability, starch is a promising candidate for replacing petroleum-based polymers [67]. Lotus seed starch, with its high amylose content and distinctive C-type crystalline structure, has advantages for fabricating functional starch nanoparticles (SNPs).

Enzymatic hydrolysis (EH), which utilizes the specific cleavage activity of enzymes, is an environmentally friendly nanofabrication technique. Nanosized starch particles have been successfully prepared by debranching or hydrolyzing lotus seed starch using pullulanase, β-amylase, and α-amylase [16]. Among these, pullulanase treatment achieved the highest conversion efficiency (81.74%), and the resulting SNPs exhibited the smallest average particle size (334.7 nm) and the highest relative crystallinity (65.07%). This result is attributed to the specific hydrolysis of α-1,6-glycosidic bonds by pullulanase, which efficiently removes branched structures in amorphous regions while preserving dense crystalline domains.

Despite the promising results of single EH treatment, SNPs obtained by this method still fall short of the requirements of high-end food industry requirements in terms of dispersion and thermal stability. To overcome these limitations, two synergistic processes—ultrasound-assisted enzymatic hydrolysis (U-EH) and high-pressure homogenization-assisted enzymatic hydrolysis (HPH-EH)—have been developed. Under optimal U-EH conditions (600 W ultrasonic power, 15 min, 3% (*w*/*w*) starch concentration), the relative crystallinity of SNPs increased to 74.92%, owing to ultrasonic cavitation enhancing enzyme-substrate contact and promoting crystallite densification [68]. With HPH-EH treatment (150 MPa, 5 cycles, 3% (*w*/*w*) starch suspension), the particle size was further reduced to 57.6 nm, with a relative crystallinity of 77.22%. This improvement is likely due to strong shear and impact forces during HPH, which effectively exfoliated lamellar structures from starch granules and generated smaller nanocrystals [68].

Acid hydrolysis and ultrasound-assisted acid hydrolysis were also effective for preparing lotus seed SNPs. The application of ultrasound shortened the preparation period by 2 days compared with conventional acid hydrolysis, significantly improving production efficiency. Optimal conditions were identified as follows: 30 min of ultrasound at 200 W combined with 5 days of acid hydrolysis yielded SNPs with the smallest particle size (147 nm) and lowest molecular weight (3.42 × 10^4^ Da), whereas 30 min of ultrasound at 150 W combined with 3 days of acid hydrolysis produced SNPs with the highest relative crystallinity (52.8%) [69].

Overall, the multiscale structure of lotus seed SNPs can be tailored by regulating enzymatic hydrolysis, physical-field-assisted processing, and acid hydrolysis. Among these, HPH-EH produces SNPs with smaller particle size and higher crystallinity compared with native starch and other modification approaches. As novel functional ingredients, carriers for the delivery of bioactive compounds, and biodegradable plastic fillers, these SNPs with tailored structural properties are expected to find applications in the food, pharmaceutical, and packaging industries.

### 5.3. Lotus Seed Resistant Starch

**Figure 4:**
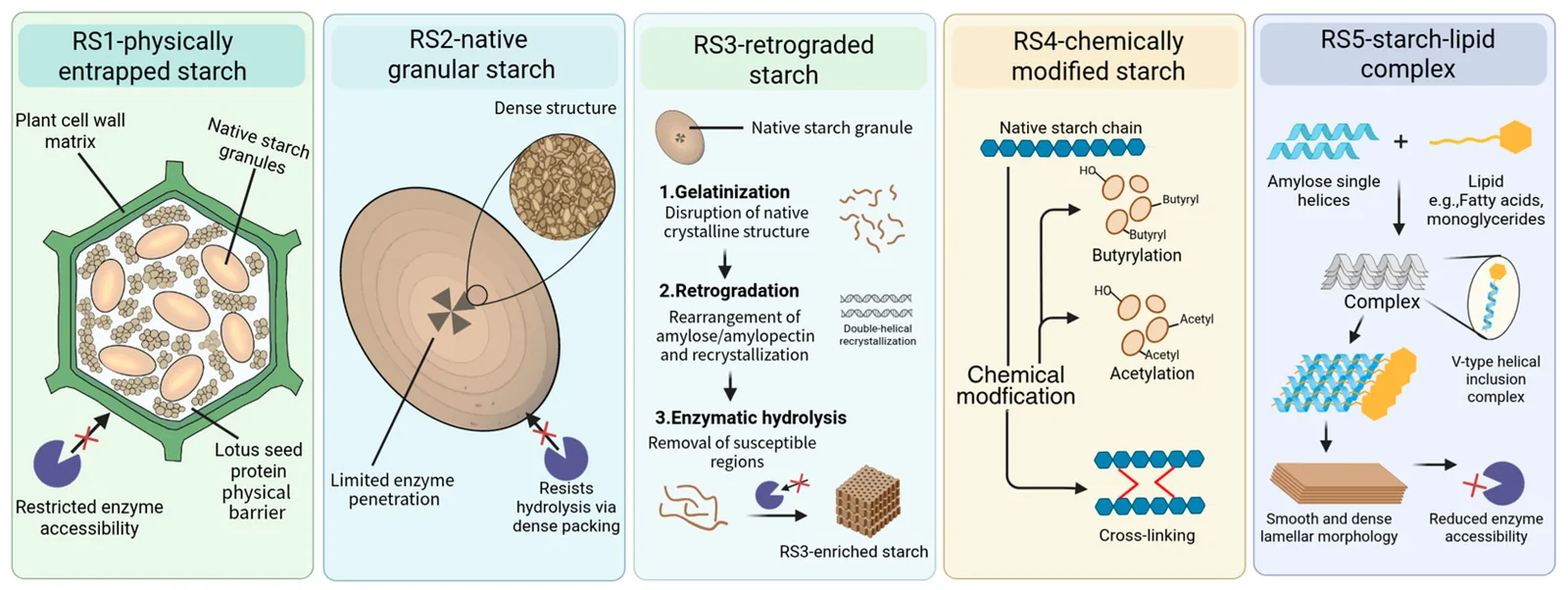
Major subtypes of lotus seed RS.

Based on differences in digestion kinetics, starch is classified into three categories: rapidly digestible starch (RDS), slowly digestible starch (SDS), and resistant starch (RS). The digestive properties of starch are inherently determined by its multiscale structural features, including crystalline polymorph, crystallinity, distribution and content of non-starch components, and the synergistic interactions among various structural attributes [70]. According to different enzyme resistance mechanisms and formation sources, RS is traditionally divided into five subtypes (Figure 4): RS1 (physically entrapped starch) refers to starch granules physically enclosed by plant cell walls or protein matrices, which prevent digestive enzymes from effectively accessing the substrate; RS2 (native granular starch) refers to naturally occurring ungelatinized starch granules that resist enzymatic hydrolysis via their ordered and dense crystalline structure; RS3 (retrograded starch) refers to ordered double-helical structures formed by the recrystallization of gelatinized starch upon cooling, which exhibit higher thermal stability and enzymatic resistance; RS4 (chemically modified starch) refers to starch derivatives obtained by altering the molecular structure through chemical modifications such as cross-linking, esterification, and etherification; RS5 (starch–lipid complex) refers to RS generated by the formation of V-type helical inclusion complexes between amylose and lipid molecules such as fatty acids and monoglycerides, which is widely present in processed foods containing lipids, such as baked goods and extruded products [71]. To date, three major subtypes of lotus seed RS, specifically RS3, RS4, and RS5, have been successfully prepared. In contrast, RS1 and RS2 are only present in small amounts in native lotus seed starch, and their resistance mainly relies on the integrity of native granules; once subjected to grinding or thermal gelatinization, their anti-digestive properties are substantially diminished [72]. In a mouse study, lotus seed RS was shown to resist digestion in the stomach and small intestine and reach the colon intact, where it promoted the proliferation of beneficial intestinal bacteria, thereby helping to regulate postprandial blood glucose and serum lipid levels in mice [73]. Whether these effects extend to humans remains to be established.

Lotus seed starch, with its high amylose content and pronounced retrogradation behavior, is a promising raw material for preparing RS3-type resistant starch. The fabrication mechanism depends on the rearrangement of amylose molecules at low temperatures after gelatinization, which facilitates the formation of a stable B-type double-helical crystalline structure. Compared with A-type crystals, such B-type crystalline structures possess better resistance to enzymatic hydrolysis. The main preparation methods of lotus seed RS include the enzymatic method [38], microwave method [17], heat-moisture treatment [19,29], and HHP treatment [22,29]. Notably, discrepancies in test methodologies and experimental parameters across different studies lead to variations in the reported RS content of lotus seed starch; thus, these literature data are only for qualitative reference (Table 2). To eliminate the interference of heterogeneous experimental conditions and ensure evaluative accuracy, this study compared RS content data derived from identical experimental systems to systematically assess the efficacy of different preparation methods. Currently, the combined “gelatinization–retrogradation–enzymolysis” approach is the mainstream process for preparing lotus seed starch with high RS content. The underlying mechanism of this integrated strategy is that physical treatments disrupt the granular structure and original crystalline order of starch, while subsequent enzymatic hydrolysis removes the amorphous regions of starch molecules and reduces molecular weight, thereby significantly enriching the ordered crystalline domains [9]. Previous studies indicate that HHP combined with enzymatic modification can maximize the RS content of lotus seed starch (up to 56.50%). After modification, lotus seed starch exhibits distinct structural changes: increased particle size, rough granule surfaces with irregular lumps, the disappearance of the Maltese cross pattern, and significantly reduced relative crystallinity and short-range molecular order [29]. In-depth analysis of the structure-function relationship reveals that the digestion resistance of lotus seed RS3 is significantly correlated with amylopectin chain length distribution, short-range ordered structure, and crystalline structure. In particular, longer amylose chains (DP > 100) and B1 chains (DP 13–24) promote the ordered molecular aggregation and subsequent formation of RS3 crystals [29].

RS4-type resistant starch is generally obtained by altering the molecular structure of starch through covalent modifications such as sodium trimetaphosphate cross-linking, esterification, and etherification, and can retain the C-type crystalline characteristics of native starch during modification. In the cross-linked lotus seed resistant starch studied by Li et al. (2022) [37], the increasing of cross-linking significantly elevated the gelatinization temperatures (*T_o_*, *T_p_*, *T_c_*) but decreased the Δ*H_gel_*, probably due to the more complex and amorphous internal structures of cross-linked starches. It should be noted that another study reported that the apparent Δ*H_gel_* of cross-linked starch composite films increased with increasing degree of cross-linking, which may be attributable to the restricted water availability during film formation and interactions between starch and other film-forming components. Because this system is not a pure starch system, these results are not directly comparable [37,52]. As the degree of cross-linking increases, the contents of RDS and SDS in the samples gradually decrease, while the RS content increases simultaneously. During the cross-linking reaction, phosphorylation reagents preferentially bound to the amorphous regions of starch and alter the original structure of starch, resulting in reduced crystallinity, swelling power, and solubility of starch, ultimately resulting in a decreased proportion of RDS and SDS and an increased proportion of RS [37,52].

RS5-type starch–lipid complexes are formed as V-type single-helical inclusion complexes between the helical cavities of amylose and lipid molecules [74]. In particular, following dynamic high-pressure microfluidization (DHPM, also referred to as high-pressure microfluidization (HPM) in some studies) treatment, the starch–lipid complexes lost their native C-type crystalline structure, accompanied by an increase in the relative intensity of V-type diffraction peaks. The newly formed V6-type polymorph featured a more tightly packed lamellar crystalline architecture with a repeating distance of 12.3 nm. This elevated structural compactness, coupled with better thermal stability, significantly restricted digestive enzyme accessibility to starch chains, thus diminishing enzymatic hydrolysis and overall digestibility. These findings demonstrate a viable continuous manufacturing approach for RS by forming complexes with short-chain fatty acids, rendering it well-suited for microwave-based and high-temperature-processed food applications [74].

It is worth noting that the digestibility of native starch is not solely determined by its microstructure; coexisting endogenous substances such as proteins also exert significant effects. For example, during digestion, the structure of lotus seed protein (LP) changes and forms a physical barrier around starch granules, thereby partially retarding starch digestion [75]. Accordingly, in the preparation of RS via modification technologies, in addition to focusing on the structural modification of starch itself, it is necessary to investigate the influence of modification methods on the material composition of starch systems. Current research on lotus seed RS mainly focuses on the RS3 subtype. Future research priorities will include the development of composite modification technologies, the analysis of structure-function relationships of different types of RS, and the expansion of their applications in the food and pharmaceutical fields.

### 5.4. Active Substance Encapsulation Carriers

The unique three-dimensional conformation and high amylose content of lotus seed starch make it a promising carrier for encapsulating active substances. Its abundant amylose fraction provides a relatively large amount of hydrophobic helical cavities, which not only enhance the formation of starch–ligand inclusion complexes, but also improve encapsulation efficiency and loading capacity [9]. To further strengthen these host-guest interactions, novel physical modification technologies such as HPH, ultrasound-microwave synergistic treatment (UM), DHPM, and HHP have been applied in the preparation of inclusion complexes [76].

Compared with conventional high-temperature gelatinization, these physical-field treatments differ in energy demand and provide non-thermal effects. The shear and cavitation forces generated by these fields effectively depolymerize amylopectin side chains and release more free linear amylose molecules, thereby increasing the number of helical sites available for inclusion. This structural reorganization promotes the complexation of starch with ligands such as lipids, polyphenols, and probiotics, endowing the resulting products with broad application prospects in food stabilization, controlled drug release, and intelligent packaging [9]. As summarized in Table 3, lotus seed starch-based carriers have been prepared with various core materials, including functional lipids, phenolic acids, vitamins, and probiotics, using different processing conditions, and their encapsulation results vary depending on the starch source, ligand type, and process parameters.

#### 5.4.1. Functional Lipid Complexes

Under appropriate conditions, lotus seed amylose can fold into a V-type single-helical conformation with hydrophobic cavities and encapsulate guest molecules via non-covalent interactions, including van der Waals forces and hydrogen bonding. This host-guest behavior renders lotus seed starch a promising delivery carrier for functional lipids and other bioactive substances [9]. For example, after ultrasound treatment at 360 W, the complexing index (CI) of V-type lotus seed starch with butyric acid reaches 28.4%. Formation of the inclusion complex increases starch crystallinity, inhibits amylose retrogradation, and raises the resistant starch content by 12.4% compared with untreated starch, while conferring improved thermal stability and hydrophobicity [77].

Fatty acid chain length is a key determinant of inclusion efficiency. Complexes of lotus seed starch with six saturated fatty acids of different chain lengths were prepared using DHPM. Short-chain fatty acids favored complex formation with lotus seed starch, whereas long-chain fatty acids reduced the CI value. The lotus seed starch–caprylic acid (C8:0) complex exhibited the highest CI of 86.3% [74]. Microwave treatment also promoted complexation between lotus seed starch and palmitic acid via hydrophobic interactions, yielding a CI of 55.41%. Thermal analysis indicates that this V-type complex primarily exists as single-helical aggregates with higher thermal stability and dissociates at 100–105 °C [78].

For heat-sensitive polyunsaturated fatty acids, DHPM also offers particular advantages. With lotus seed starch as a carrier, inclusion complexes of lotus seed starch with linolenic acid prepared at 180 MPa exhibit higher crystallinity and improved thermal stability, with a linolenic acid loading capacity of 14.84 mg/g (approximately 1.48%, *w*/*w*). Complexation decreases the short-range molecular order of starch, increases its room-temperature solubility, and raises the RS content to 17.42% [79,91]. CLA, another heat-sensitive lipid, can be complexed with lotus seed starch after HHP treatment. Lotus seed starch is first modified by OSA esterification combined with HHP, and the modified starch is then complexed with CLA to form an OSA-LS-CLA ternary complex. Hydrophobic groups introduced by OSA enhance intermolecular interactions between starch and CLA, while HHP promotes amylose chain unfolding and the formation of hydrophobic cavities, facilitating CLA encapsulation. This composite structure showed thermal stability comparable to that of native lotus seed starch (Δ*H_gel_*: 9.98 vs. 10.40 J/g). Compared with native lotus seed starch, it gave a 26% lower glucose release after 180 min of in vitro digestion (16.93 vs. 22.95 mg/mL), indicating enhanced resistance to enzymatic digestion [39].

In emulsion systems, DHPM treatment of starch–lecithin complexes (90 MPa) produces complexes with a CI of 41.6%. Their molecular architecture inhibits amylopectin degradation and amylose retrogradation. Notably, the crystalline polymorph evolves with increasing homogenization pressure: the crystalline pattern transitions from V6-II to V6-I and then reverts to V6-II [80].

Overall, the formation efficiency and properties of lotus seed starch–lipid complexes are co-regulated by fatty acid chain length, processing method, and process parameters. By judiciously selecting physical-field-assisted methods, researchers can obtain functional ingredients with higher crystallinity, improved thermal stability, and defined V-type crystalline structures, providing a theoretical basis for developing nutrient delivery carriers and functional food ingredients.

#### 5.4.2. Vitamin and Polyphenol Complexes

Encapsulation with lotus seed starch effectively protects oxidation- and degradation-prone nutrients such as polyphenols and vitamins. Endogenous phenolic acids and other phytochemicals in lotus seeds can spontaneously bind to native lotus seed starch surfaces via physical interactions dominated by hydrogen bonding, van der Waals forces, and hydrophobic effects [92]. Chlorogenic acid, and *p*-hydroxybenzoic acid form single-helical inclusion complexes with starch, whereas coumaric acid forms non-inclusion complexes. Adsorption of those phenolic acids increases the total color difference (Δ*E*), particle size, zeta potential, and Δ*H_gel_* of starch granules, while decreasing bound water content, glass transition temperature (*T_g_*), and loss modulus (*G″*) of starch paste. Reduced bound water content leads to lower storage modulus (*G′*) and diminished retrogradation of starch paste [92].

Under different processing conditions, lotus seed starch–green tea polyphenol (LS-GTP) complexes prepared by HPH, HHP, and UM show significant differences in yields and structural characteristics [83,93]. When GTPs (10% *w*/*w* based on starch) are added and the suspension is treated by HPH, 150 MPa emerges as the critical pressure for structural transition: below this threshold, hydrogen-bonded non-inclusion complexes with C-type crystals and reticulated surfaces form; above it, hydrophobic inclusion complexes with V-type crystals and flower-like surfaces dominate [83].

In the chlorogenic acid-lotus seed starch system, low-pressure treatment (static 100 MPa/dynamic 10 MPa) induces non-inclusion complexes. When the pressure increases to static 500 MPa/dynamic 50 MPa, non-inclusion complexes largely disappear and inclusion complexes predominate. High pressure disrupts starch granule barriers, dissociates double helices, and releases starch molecular chains, thereby facilitating penetration of chlorogenic acid and promoting the assembly of both inclusion and non-inclusion complexes driven by hydrophobic interactions. The inclusion rate in the dynamic high-pressure group (50 MPa) reaches 26.01%, higher than 15.32% in the static high-pressure group (500 MPa) [81].

Comparative studies on heat treatment (HT), UM, DHPM, and HHP reveal that both DHPM- and UM-treated lotus seed starch–EGCG (LSE) complexes form denser structures and exhibit reduced hydrolysis rates; among these, LSE-DHPM shows the strongest resistance to enzymatic hydrolysis, whereas LSE-HT shows the weakest [40,93]. Complexes formed on starch granule surfaces act as physical barriers, hindering contact between digestive enzymes and substrates. LS-EGCG complexes prepared by different processes all exhibit sustained release of EGCG, but release profiles differ markedly among treatments; notably, HPM produces a denser starch structure with the slowest overall release in simulated gastrointestinal conditions [82]. In addition, chlorogenic acid released during α-amylase hydrolysis can further retard starch digestion by inhibiting α-amylase activity, potentially contributing to improved glycemic control [81].

Vitamin C and vitamin E (VC and VE) are natural antioxidants that participate in numerous biochemical reactions and play important roles in preventing cancer and cardiovascular diseases, reducing adverse pregnancy outcomes, and enhancing immune function. Their interactions with starch can modify both morphological and functional properties of starches [84]. Microwave treatment alone disrupts the short-range ordered structure of lotus seed starch, reduces its granular thermal stability, and increases the apparent amylose content; these effects become progressively more pronounced as microwave power and treatment time increase. VC mitigates this structural deterioration and thermal instability, increases Δ*H_gel_*, while significantly decreasing pasting viscosity; its complexing rate with starch ranges from 76.78% to 90.84% as reported in an unpublished Master’s thesis [85].

Mechanistically, low concentrations of VC can induce starch chain scission and alter molecular weight and particle size via hydrogen bonding. As vitamin C concentration increases, its protective effect on ordered structures becomes more pronounced, and the resulting densified structure ultimately enhances the thermal stability and digestion resistance of starch while inhibiting short-term retrogradation [85]. VE-lotus seed starch complexes can be prepared using three non-thermal treatments: HPH, HHP, and freeze–thaw treatment. After a single freeze–thaw cycle, the complexing rate of VE reaches 59.66%. Infrared spectroscopy indicates the formation of weak hydrogen bonds between VE and starch, which enhance short-range order and promote the formation of double-helical structures. Concurrently, hydrophobic interactions promote molecular entanglement, increase gel hardness, and mitigate the loss of crystallinity caused by processing, as reported in an unpublished Master’s thesis [86].

VE can also improve the short-range order and thermal stability of microwave treated starch, although this effect is only pronounced at low VE concentrations. In contrast to VC, VE reduces amylose content and increases pasting viscosity. Its complexing rate with starch ranges from 44.16% to 81.49%, lower than that of VC in the same study [85]. VE significantly decreases the proportion of amorphous regions and promotes helical ordering, thereby enhancing thermal stability and lowering digestibility via the formation of denser structures [85].

In summary, VC and VE exert opposite regulatory effects on the physicochemical and processing properties of lotus seed starch at the multiscale structural level, primarily through hydrogen bonding and hydrophobic interactions, respectively.

#### 5.4.3. Probiotic Delivery Carriers

Probiotics exert diverse physiological functions and play a key role in regulating intestinal microecology and maintaining host health. However, they are highly sensitive to environmental stresses. Following oral administration, probiotics are easily inactivated by harsh gastrointestinal conditions, which greatly limits their efficacy. Microencapsulation is therefore widely used to enhance the environmental tolerance and gastrointestinal survival of probiotics [94]. Native lotus seed starch, due to its low specific surface area and limited structural stability, is less suitable as a wall material for probiotic encapsulation, and thus requires modification to optimize its structure and functional properties [87].

Lotus seed porous starch (LPS) has been prepared by combined enzymatic hydrolysis using α-amylase and glucoamylase, and used together with sodium alginate as a composite wall material to encapsulate *Lactobacillus acidophilus*. Under optimized conditions, the encapsulation efficiency reached 80.16%. Compared with free cells, the microcapsules showed improved heat tolerance. After 2 h in simulated gastric fluid, viable counts remained above 10^7^ CFU/g, indicating an effective acid-resistant barrier. After 4 h in simulated intestinal fluid, the encapsulated bacteria were fully released, with viable counts of 7.56 log CFU/g, demonstrating good intestinal targeted-release performance, as reported in an unpublished Master’s thesis [89].

The rough grooves and cavity structures on the surface of lotus seed resistant starch can provide abundant adhesion sites for *Bifidobacterium*. Microcapsules constructed from lotus seed resistant starch and *B. adolescentis*, effectively reduce bacterial loss in simulated gastric juice, exhibit good solubility in intestinal fluid, and improve the storage stability and stress resistance of *B. adolescentis*, as documented in an unpublished Master’s thesis [90]. Probiotic microcapsules prepared with cross-linked lotus seed resistant starch–sodium alginate (LC-SA) as composite wall materials can also function as an efficient oral delivery system. A higher degree of cross-linking in lotus seed resistant starch gradually improves encapsulation efficiency, heat tolerance, tolerance to simulated gastric fluid, and performance during sequential gastrointestinal digestion and release [87,88].

In summary, lotus seed starch modified by enzymatic porosification, resistant starch formation, and cross-linking can be combined with sodium alginate to construct microcapsule carriers with improved encapsulation efficiency and intestinal targeting compared with free probiotics or unmodified starch-based carriers. These approaches offer multiple technical routes for probiotic encapsulation and intestinal targeted delivery.

### 5.5. Lotus Seed Starch as a Food Ingredient

Because of its distinctive chemical composition and multiscale structural features, lotus seed starch combines a high amylose content, a C-type crystalline polymorph, good gelation capacity but relatively weak gel strength, and modifiable digestibility, making it a natural raw material and functional food ingredient.

Reconstituted rice has been produced by extrusion using mixtures of lotus seed starch and broken rice flour at different ratios. The optimal ratio was determined by assessing the pasting properties of the mixed flours, together with the sensory quality, texture profile, and cooking loss of the reconstituted rice. Extrusion parameters (feed moisture, die temperature, screw speed) were optimized by response surface methodology. With increasing lotus seed starch content, all viscosity indices of the mixed flour and the cooking loss of reconstituted rice increased. Sensory scores, springiness, chewiness, and cohesiveness first increased and then decreased, whereas hardness increased continuously. Overall quality was optimal at a lotus seed starch addition level of 30%. The optimal extrusion conditions were a feed moisture of 40%, screw speed of 210 r/min, and die temperature of 95 °C [95].

In a rat study, compared with conventional rice, reconstituted rice containing a high proportion of lotus seed starch was shown to alleviate lipid metabolism disorders and lipid accumulation induced by a high-fat diet. More than 20 differentially abundant lipid metabolites, including phosphatidylcholine (PC), triglycerides (TG), and ceramides (Cer), were identified, indicating substantial remodeling of lipid metabolism [96]. These findings suggest a potential benefit for human health, but clinical evidence is currently lacking.

## 6. Conclusions and Perspectives

Lotus seed starch is the most abundant component in lotus seeds. Although lotus seeds have a long history of use in traditional Chinese foods, systematic fundamental research and industrial development specifically targeting lotus seed starch remain limited. This review systematically summarizes the multiscale structural characteristics and key physicochemical properties of lotus seed starch; moreover, it comprehensively reviews recent advances in major modification strategies and application directions. However, it is noted that the industrial application of modified lotus seed starch remains unrealized, and future research should prioritize the directions summarized in Table 4.

The multilevel compositional and structural heterogeneity of lotus seed starch provides a material basis for diversified applications. Meanwhile, to mitigate the inherent defects of lotus seed starch, various methods have been employed for its modification. In particular, physical modification technologies—including microwave treatment, HPH, UM, and HHP—are becoming research hotspots, as they enabling potential modulation of starch structure across multiple scales. Modified lotus seed starch with improved thickening, stabilizing, and gelling properties has found potential applications in edible packaging films, nanomaterials, low-GI functional foods, and sustained-release carriers for bioactive compounds, thereby highlighting its considerable industrial potential.

However, due to poor raw material consistency, difficulty in regulating the microstructure, and other factors, the modification of lotus seed starch is largely limited to laboratory studies, lacking a scalable, industrialized technological framework. Most reported modification studies are at laboratory scale (gram to kilogram quantities). The use of modified lotus seed starch as a commercial starch ingredient has not yet been realized. Commercial applications have been achieved primarily in lotus seed food products, where starch modification technologies have enabled the development of canned lotus seeds, ready-to-eat lotus products, and lotus fruit pastries. Future research should include pilot-scale validation and techno-economic analysis to determine which modification methods are truly scalable and economically viable (Table 4).

The economic feasibility of lotus seed starch as a bulk commodity is limited by the high cost of lotus seeds compared with established commercial starches such as corn, potato, and cassava. Therefore, its industrial potential should be oriented toward high-value niche applications, including resistant starch for prebiotics, functional food ingredients, and encapsulation carriers, where its unique structural properties (high amylose content, C-type crystallinity) provide specific advantages. Compared with corn starch and potato starch, which have already established mature starch-based biomaterial systems, lotus seed starch faces several challenges, including low encapsulation efficiency of active compounds, poorly controlled release kinetics, inadequate mechanical properties of the derived materials, and difficulties in scaling up production. Concurrently, the biological and efficacy evaluation frameworks for lotus seed starch remain inadequate. Current research is predominantly limited to in vitro cellular tests and rudimentary animal studies, suffering from a critical absence of standardized, systematic in vivo biological assessments. This gap prevents the comprehensive verification of material biocompatibility, in vivo degradation profiles, and integrated therapeutic efficacy.

## Figures and Tables

**Table 1:** Modification of lotus seed starch and lotus seed flour.

Modification Method	Substrates	Process Conditions	Structural Change	Physicochemical Effects	Functional Consequence	Potential Application	References
Physical Modification							
Heat-Moisture Treatment	Isolated starch	The starch was adjusted to a moisture content of 30% (*w*/*w*), equilibrated at 25 °C for 24 h, and heated at 110 °C for 8 h, and then vacuum dried at 45 °C for 24 h	Increased short-range molecular order and double-helix organization; altered granular morphology	Increased gelatinization temperatures and enthalpy.	Increased gel elasticity and adhesiveness	Gelling, thickening, stabilizing and filling agents for starch-based food formulations	[19]
	Whole flour	Lotus seed flour was mixed with deionized water at a solid-to-liquid ratio of 1:4 (*w*/*v*), and equilibrated overnight at 4 °C. The mixture was then hot-air dried at 40 °C for 12–14 h to a moisture content of 25% (*w*/*w*), followed by treatment at 100 °C for 2 h	Increased apparent amylose content; restricted granular swelling	Reduced swelling power and lower pasting viscosity than native flour	Resistant starch content was elevated.	Low-GI flour ingredient	[31]
Dry Heat Modification	Isolated starch	Starch (19.8 g) was dispersed in 40 mL of distilled water. The mixture was stirred for 30 min until homogeneous, adjusted to pH 7, and dried at 45 °C for 13 h. The dried mixture was then ground, passed through an 80-mesh sieve, and heated at 130 °C for 3 h	Slight surface cracks	Increased solubility and pasting temperature; decreased swelling power	Reduced retrogradation and syneresis	Starch-based biodegradable films	[32]
High Hydrostatic Pressure	Isolated starch	A 15% (*w*/*w*) aqueous starch suspension was subjected to high-pressure treatment at 25 °C for 30 min under pressures ranging from 100 to 600 MPa, with a pressurization rate of 1 MPa/s	Loss of polarization cross at 600 MPa (partial gelatinization); reduced crystallinity; pressure-induced granular disruption	Shear-thinning (pseudoplastic) behavior; consistency coefficient increased while yield stress and flow index decreased	Gel structure strengthened at 100–500 MPa but weakened at 600 MPa; lower recrystallization rate during storage	Starch gels requiring reduced retrogradation	[28]
High-Pressure Homogenization	Isolated starch	A 3% (*w*/*v*) starch suspension was homogenized under 0–180 MPa for 15 min	Destruction of both crystalline and amorphous regions; reduced short-range order; C-to-B crystalline transition, and granular fragmentation	Reduced transition temperatures, enthalpy, and pasting viscosities; increased swelling power and solubility	Increased digestion efficiency	Rapidly digestible starch-based formulations	[33]
Ultrasonic Treatment	Isolated starch	A 30% (*w*/*v*) starch suspension was sonicated at 20 kHz, 170 W and 20 °C for 30 min. After cooling to room temperature, it was centrifuged at 6000× *g* for 15 min, and the pellet was hot-air dried at 45 °C for 12 h	Surface cracks; altered granular organization	Increased swelling power, solubility, apparent amylose content, and gelatinization enthalpy	Enhanced hydration capacity and granular thermal stability	Starch formulations requiring enhanced hydration	[34]
Microwave Treatment	Isolated starch	A 30% (*w*/*v*) lotus seed starch suspension was heated at gradient microwave powers of 2.4, 4.0, 6.4 and 8.0 W/g until complete gelatinization	Enhanced double-helix organization and amylose–amylose interactions; reduced branching degree of amylopectin; reduced molecular size and radius of gyration	Reduced swelling power and amylose leaching	The contents of resistant starch and slowly digestible starch increased significantly; reduced hydrolysis index (HI) and glycemic index (GI)	Low-GI starch ingredient; formulations with controlled digestibility	[17]
Irradiation	Isolated starch	Packaged lotus seed starch samples were subjected to ^60^Co γ-irradiation at 23 °C at four doses (5, 10, 15, 20 kGy)	Bimodal granule size distribution; reduced relative crystallinity; minor surface roughening at 15–20 kGy	Reduced amylose content, gelatinization temperatures, and pasting viscosities	Markedly lower processing viscosity; reduced retrogradation tendency	Starch formulations requiring lower processing viscosity and reduced retrogradation	[20]
Chemical Modification							
Acetylation	Isolated starch	Acetic anhydride was added at 0%, 3%, 6%, and 12% (dry weight of starch) to a 30% (*w*/*w*) starch suspension at pH 8.0–8.4, followed by a further 0.5 h reaction	Introduction of acetyl groups; decreased relative crystallinity without changing the crystalline type; increased surface roughness	Increased particle size, transparency, swelling power, and solubility; reduced gelatinization enthalpy	Enhanced hydration capacity and paste clarity/transparency	Starch formulations requiring improved hydration and transparency	[35]
Butyrylation	Isolated starch	Butyric anhydride (0.125–8 times starch dry weight) was added dropwise to a 30% (*w*/*v*) starch suspension at pH 8.0–8.5, followed by a 4 h reaction at 40 °C	Introduction of butyryl groups at C2, C3, and C6; reduced molecular weight and crystallinity; rougher and more broken granule surface at high DS	Reduced gelatinization enthalpy and final viscosity	Increased resistant starch (RS) content	Resistant starch ingredient	[36]
Cross-Linking	Isolated starch	Lotus seed starch (20 g) was dispersed in 30 mL water with 2 g sodium sulfate, adjusted to pH 11.5, mixed with 1–12% (*w*/*w*, starch basis) blended cross-linking agent (STMP/STPP = 99:1), and reacted at 50 °C for 4 h. The pH was then adjusted to 6.5, and the product was hot-air dried at 45 °C for 24 h	Formation of phosphate cross-links, predominantly distarch monophosphate; increased granular agglomeration	Reduced solubility and increased paste stability under heating and shear	Cross-linking elevated resistant starch content	Heat-/shear-stable starch matrix and resistant starch ingredients	[37]
Acid Treatment	Isolated starch	Purified native lotus seed starch (100 g) was mixed with 0.2 mol/L citric acid solution, adjusted to 30% wet-basis moisture, sealed, equilibrated at 25 °C for 24 h, heated at 110 °C for 8 h, and finally neutralized	Increased short-range molecular order and double-helix organization	Increased gelatinization temperatures and enthalpy	Gel elasticity and adhesiveness were better than those of native starch	Gelling, thickening, stabilizing and filling agents for starch-based formulations	[19]
Enzymatic Modification							
Pullulanase	Whole flour	A 10% (*w*/*v*) native lotus seed flour suspension in deionized water was treated with pullulanase at 20 NPUN/g dry flour, and reacted at pH 4.5 and 55 °C for 6 h	Enzymatic debranching of amylopectin; increased apparent amylose content	Increased pasting viscosity and accelerated paste development	Increased resistant starch (RS) content	Low-GI functional flour formulations	[31]
α-Amylase and Glucoamylase	Isolated starch	A 30% (*w*/*w*) starch suspension was treated with 0.5–2.5% (*w*/*w*, starch basis) composite enzyme (α-amylase/glucoamylase = 1:2, *w*/*w*; 50,000 and 100,000 U/g, respectively) at pH 5.5 and 55 °C for 12 h	Formation of surface pores; increased specific surface area and pore volume; increased relative crystallinity and ordered structure; reduced particle size	Increased water and oil absorption capacities	Elevated SDS and resistant starch levels.	Adsorption and encapsulation matrices; resistant or slowly digestible starch ingredient	[38]
Dual Modification							
Octenyl Succinic Anhydride (OSA) Esterification Combined with High Hydrostatic Pressure	Isolated starch	A 35% (*w*/*w*) starch suspension reacted with 7% (starch weight basis) octenyl succinic anhydride (OSA) at pH 8.5 and 35 °C to obtain OSA-modified lotus seed starch. Then 10 g of the modified starch was dispersed in 90 mL absolute ethanol, mixed with conjugated linoleic acid, and treated at 600 MPa and 25 °C for 30 min	Formation of OSA-LS-CLA complexes; increased crystallinity and molecular organization	Improved complex thermal stability; enhanced swelling power and solubility with increasing temperature	The OSA-LS-CLA complex showed a reduced digestion rate (26% lower than native LS)	Starch–lipid ingredients with reduced digestibility	[39]
Ultrasound-Microwave (UM) Combined Synergistic Treatment	Isolated starch	The lotus seed starch–green tea polyphenol (LS-GTP) physical mixture was treated with combined ultrasound (200–1000 W) and microwave (175 W) for 15 min	Formation of V-type starch–polyphenol inclusion complexes and non-inclusion complexes through molecular interactions	Altered granule morphology and XRD pattern; effects stronger than either treatment alone	Reduced microwave energy consumption and increased complex yield; reduced digestion efficiency	Starch–polyphenol systems with reduced digestibility	[40]
HMT-Ultrasound Combined Synergistic Treatment	Isolated starch	Lotus seed starch was adjusted to 30% (*w*/*w*) moisture, equilibrated at 6 °C overnight, heated at 110 °C for 3 h, cooled and dried at 40 °C for 24 h. The dried starch was formulated into a 30% (*w*/*v*) aqueous suspension and sonicated at 20 kHz, 170 W and 20 °C for 30 min	Increased surface cracking and granular structural disruption compared with single treatment	Reduced swelling power, solubility, amylose content, and peak viscosity; increased gelatinization enthalpy; gels elastic	Increased resistant starch content, and decreased slowly digestible starch content.	Weaning food components; resistant starch ingredients	[34]

**Table 2:** Preparation methods of resistant starch from lotus seeds and their content.

Preparation Methods	Process Conditions	Resistant Starch Content	Reference
Enzymatic Debranching	Debranching with pullulanase (20 U/g starch) at 55 °C for 12 h, then incubation at 4 °C for 24 h, followed by freezing at −18 °C overnight.	46.49%	[29]
	Treatment with thermostable α-amylase (5000 U/g) at 95 °C, pH 6.0 for 1 h, then with glucoamylase at 60 °C, pH 4.5 for 1 h.	41.89%	[22]
	A 30% (*w*/*w*) lotus seed starch suspension was adjusted to pH 5.5 and pre-incubated at 55 °C for 15 min. A composite enzyme (α-amylase:glucoamylase = 1:2, *w*/*w*) was added at 1.5% (*w*/*w*, based on starch), corresponding to 250 U/g starch of α-amylase and 1000 U/g starch of glucoamylase, and hydrolysis at 55 °C for 12 h.	Approximately 70%	[38]
Autoclaving Treatment	Autoclaved at 121 °C for 60 min, refrigerated at 4 °C for 24 h, then frozen at −18 °C overnight.	35.77%	[29]
High Hydrostatic Pressure	High-pressure processed at 600 MPa and room temperature for 30 min, refrigerated at 4 °C for 24 h, then frozen at −18 °C overnight.	29.82%	[29]
Microwave Treatment	Microwave-irradiated at 490 W for 120 s, refrigerated at 4 °C for 24 h, then frozen at −18 °C overnight.	40.42%	[29]
	Microwave-irradiated at 640 W for 2 min, then retrograded at 4 °C for 24 h.	39.53%	[22]
	Microwave-heated at 8.0 W/g until complete gelatinization.	30.42%	[17]
Autoclaving Combined with Enzymatic Hydrolysis	Debranched with pullulanase (20 U/g, 55 °C, 12 h), enzyme inactivated (100 °C, 15 min), then autoclaved (121 °C, 60 min), refrigerated (4 °C, 24 h), and frozen again (−18 °C, overnight).	42.28%	[29]
High Hydrostatic Pressure Combined with Enzymatic Hydrolysis	Debranched with pullulanase (20 U/g, 55 °C, 12 h), enzyme inactivated (100 °C, 15 min), then high-pressure processed (600 MPa, room temp., 30 min), refrigerated (4 °C, 24 h), and frozen again (−18 °C, overnight).	56.50%	[29]
Microwave Combined with Enzymatic Hydrolysis	Debranched with pullulanase (20 U/g, 55 °C, 12 h), enzyme inactivated (100 °C, 15 min), then microwave-irradiated (490 W, 120 s), refrigerated (4 °C, 24 h), and frozen again (−18 °C, overnight).	47.00%	[29]
Ultrasound-Coupled Autoclaving Treatment	Ultrasonicated at 25 °C, 300 W for 55 min, then autoclaved at 115 °C for 15 min, cooled to room temperature, and retrograded at 4 °C for 24 h.	56.12%	[22]
Cross-Linking Modification	Lotus seed starch (20 g) dispersed in 30 mL water with 2 g sodium sulfate, pH adjusted to 11.5, cross-linked with a 99:1 (*w*/*w*) sodium trimetaphosphate: sodium tripolyphosphate blend (1–12% *w*/*w* starch) at 50 °C for 4 h, then pH adjusted to 6.5 and hot-air dried at 45 °C for 24 h.	49%	[37]

**Table 3:** Preparation methods and properties of lotus seed active substance encapsulates.

Core Material	Process Conditions	Encapsulation Results	References
Functional lipids			
Butyric acid	5% (*w*/*v*) V-type lotus seed starch (VLS) suspension stirred at 40 °C for 30 min, then butyric acid (BA) solution added at 4× dry VLS weight and reacted at 40 °C for 4 h.	The complexation increased starch crystallinity, inhibited amylose retrogradation, imparted better thermal stability and hydrophobicity, and yielded a 12.4% higher RS content than the untreated sample.	[77]
Caprylic acid	Fatty acid (0.1 g) dissolved in 50% ethanol, mixed with 50 mL of 4% (*w*/*w*) defatted lotus seed starch slurry, then homogenized for 8 passes using a dynamic high-pressure microfluidizer at 200 MPa.	The starch–caprylic acid complex exhibited a well-defined V6-type semi-crystalline structure, higher melting temperature, and reduced digestibility.	[74]
Palmitic acid	6% (*w*/*v*) aqueous starch suspension with palmitic acid added at 2% (*w*/*w*, dry starch basis), stirred at 50 °C for 1 h, then subjected to gradient microwave power treatments (600–1000 W, 90 s each) with stirring at 120 rpm.	A complexation index of 55.41% was attained; palmitic acid promoted the transformation of amorphous starch chains into ordered single-helical structures, thereby improving thermal stability.	[78]
Linolenic acid	6% (*w*/*v*) lotus seed starch aqueous suspension with α-linolenic acid added at 6% (*w*/*w*, dry starch basis), processed through a dynamic high-pressure microfluidizer at 180 MPa for 7 cycles.	The complex exhibited higher crystallinity and thermal stability, with an ALA content of 14.84 mg/g (approximately 1.48%).	[79]
Conjugated linoleic acid	OSA-modified lotus seed starch (10 g) dispersed in 90 mL absolute ethanol, conjugated linoleic acid added and mixed, then held at 600 MPa, 25 °C for 30 min.	The complexation index was inversely correlated to the amount of conjugated linoleic acid, reaching a maximum of 81.31%.	[39]
Lecithin	5% (*w*/*w*) defatted lotus seed starch with lecithin added at 0–5% (*w*/*w*, dry starch basis) at 50 °C, then processed by a dynamic high-pressure microfluidizer at 25 °C for 5 cycles each at 60, 90, and 120 MPa.	Complexation index reached a maximum of 41.6%; the complex maintained granule integrity, inhibited retrogradation, and its crystalline type dynamically evolved with increasing homogenization pressure.	[80]
Phenolic acids			
Chlorogenic acid	Chlorogenic acid added at 6% (*w*/*w*) of dry lotus seed starch, total solids adjusted to 6% (*w*/*v*), held at 100 and 500 MPa for 30 min each, then equilibrated at 4 °C for 24 h.	Encapsulation efficiency was 15.32%; low-pressure treatment formed non-inclusion complexes, while high-pressure treatment yielded inclusion complexes.	[81]
	Chlorogenic acid added at 6% (*w*/*w*) of dry lotus seed starch, total solids maintained at 6% (*w*/*v*), homogenized at 10 and 50 MPa for 12 min, then equilibrated at 4 °C for 24 h.	Encapsulation efficiency was 26.01%; low-pressure treatment produced non-inclusion complexes, while high-pressure treatment produced inclusion complexes.	[81]
Epigallocatechin gallate (EGCG)	A suspension of lotus seed starch (10 g) and EGCG (1.0 g) in 150 mL distilled water was subjected to four separate treatments: (i) heating at 100 °C for 30 min; (ii) combined ultrasound (375 W) and microwave (300 W) under the same time conditions as heating (iii) high-pressure microfluidization at 120 MPa for 9 cycles; and (iv) HHP at 600 MPa, room temperature for 90 min.	Controlled release of EGCG, and the high-pressure microfluidics-prepared complex showed higher resistance to enzymatic hydrolysis.	[82]
Green tea polyphenols	Green tea polyphenols added at 10% (*w*/*w*, dry starch basis) to a 3% (*w*/*v*) lotus seed starch suspension, then homogenized at 60–180 MPa for 5 passes.	This composite system possessed stronger resistance to enzymatic digestion.	[83]
Vitamins			
Vitamin C	8% (*w*/*v*) aqueous starch suspension, with vitamin C added at 4% (*w*/*w*, dry starch basis) for the composite group, then microwave treatment at 2, 4, 6, and 8 W/g for 6 min each.	The complex exhibited a denser structure, inhibiting short-term retrogradation and enhancing thermal stability and resistance to enzymatic hydrolysis.	[84]
	Lotus seed starch aqueous suspensions (8%, *w*/*v*) with vitamin C added at 0.25–4% (*w*/*w*, dry starch basis), then microwave treatment at 6 W/g for 360 s.	Vitamin C complexation efficiency ranged from 76.78% to 90.84%, regulating starch molecular weight and particle size via hydrogen bonding, while improving thermal stability, enzyme resistance, and suppressing retrogradation.	[85]
Vitamin E	5% (*w*/*v*) aqueous lotus seed starch suspension, with vitamin E in absolute ethanol added at 2% (*w*/*w*, dry starch basis), magnetically stirred for 10 min, then gelatinized at 85 °C for 25 min, cooled to room temperature, and subjected to freeze–thaw cycles (one cycle: freezing at −20 °C for 22 h, thawing at 25 °C for 2 h).	Vitamin E complexation efficiency reached 59.66%, decreasing amorphous content and promoting helical ordering, thereby enhancing thermal stability and reducing digestibility.	[86]
	Lotus seed starch suspensions (8%, *w*/*v*) with vitamin E added at 0.25–2% (*w*/*w*, dry starch basis), then microwave treatment at 6 W/g for 360 s.	Vitamin E complexation efficiency ranged from 44.16% to 81.49%, improving short-range molecular order and thermal stability of microwave-treated starch (pronounced only at low concentrations), while reducing amylose content and increasing pasting viscosity.	[85]
Probiotics			
*Lactiplantibacillus plantarum* ACCC 11095	Medium- and high-cross-linked lotus seed resistant starches (LS-2/6/12CS) were combined with sodium alginate to prepare microcapsules.	The encapsulation efficiency reached 48.81%, and the viable probiotic release count exceeded 10^6^ CFU/g.	[87,88]
*Lactobacillus acidophilus*	Enzymatically hydrolyzed lotus porous starch (LPS with 1.5% enzyme addition) combined with sodium alginate as composite wall material to encapsulate *Lactobacillus acidophilus* by extrusion; optimal parameters: LPS 5%, sodium alginate 4%, wall material-to-bacteria ratio 3:1 (*v*/*v*), CaCl_2_ 3%.	Encapsulation efficiency of *L. acidophilus* reached 80.16%, enhancing heat and acid tolerance and enabling targeted intestinal release.	[89]
*Bifidobacterium adolescentis* ATCC 15703	Adhesive LRS3-*Bifidobacterium* system as core material, sodium alginate as principal wall material; microcapsules prepared via internal emulsification. Optimal parameters: CaCO_3_:SA mass ratio 1:2.9, glacial acetic acid 438.9 mL, water-to-oil volume ratio 30:109.7, emulsifier concentration 2.5%.	Encapsulation efficiency reached 75.27%, improving storage survival rate, acid tolerance, and achieving site-specific intestinal release.	[90]

**Table 4:** Main Research gaps and future directions for lotus seed starch.

Research Area	Current Research Gap	Proposed Future Directions
Raw material consistency and standardization	Poor raw material consistency; limited comparative studies across cultivars, origins, and extraction methods.	Establish standardized cultivar screening and extraction protocols; build a cultivar-structure-function database.
Microstructure regulation	Difficulty in precisely regulating the multiscale microstructure of lotus seed starch.	Develop green and precise modification technologies to achieve high uniformity, better mechanical strength, and high loading capability.
Green modification and scalability	Green modification methods are promising but largely limited to laboratory scale.	Conduct pilot-scale trials and life cycle assessment; compare with conventional modification on cost, energy consumption, and environmental impact.
Material performance	Low encapsulation efficiency, poorly controlled release kinetics, and inadequate mechanical properties.	Optimize composite modification and formulation; elucidate material–structure–function relationships and build synergistic regulation models.
Biological and efficacy evaluation	Predominantly in vitro and preliminary animal studies; lack of standardized, systematic in vivo assessments.	Conduct comprehensive in vivo studies on biocompatibility, degradation profiles, and integrated therapeutic efficacy.
Industrial implementation	Industrial application remains unrealized; lack of scalable technological framework and commercial validation.	Foster interdisciplinary collaboration; evaluate cost, regulations, and market acceptance; benchmark against established commercial starches.

## Data Availability

No new data were created or analyzed in this study. Data sharing is not applicable to this article.
